# Genomic comparison of 93 *Bacillus* phages reveals 12 clusters, 14 singletons and remarkable diversity

**DOI:** 10.1186/1471-2164-15-855

**Published:** 2014-10-04

**Authors:** Julianne H Grose, Garrett L Jensen, Sandra H Burnett, Donald P Breakwell

**Affiliations:** Microbiology and Molecular Biology Department, Brigham Young University, Provo, UT USA

**Keywords:** Bacteriophage, Phage, Cluster, *Bacillus*

## Abstract

**Background:**

The *Bacillus* genus of Firmicutes bacteria is ubiquitous in nature and includes one of the best characterized model organisms, *B. subtilis,* as well as medically significant human pathogens, the most notorious being *B. anthracis* and *B. cereus.* As the most abundant living entities on the planet, bacteriophages are known to heavily influence the ecology and evolution of their hosts, including providing virulence factors. Thus, the identification and analysis of *Bacillus* phages is critical to understanding the evolution of *Bacillus* species, including pathogenic strains.

**Results:**

Whole genome nucleotide and proteome comparison of the 93 extant *Bacillus* phages revealed 12 distinct clusters, 28 subclusters and 14 singleton phages. Host analysis of these clusters supports host boundaries at the subcluster level and suggests phages as vectors for genetic transfer within the Bacillus cereus group, with *B. anthracis* as a distant member of the group. Analysis of the proteins conserved among these phages reveals enormous diversity and the uncharacterized nature of these phages, with a total of 4,922 protein families (phams) of which only 951 (19%) had a predicted function. In addition, 3,058 (62%) of phams were orphams (phams containing a gene product from a single phage). The most populated phams were those encoding proteins involved in DNA metabolism, virion structure and assembly, cell lysis, or host function. These included several genes that may contribute to the pathogenicity of *Bacillus* strains.

**Conclusions:**

This analysis provides a basis for understanding and characterizing *Bacillus* phages and other related phages as well as their contributions to the evolution and pathogenicity of Bacillus cereus group bacteria. The presence of sparsely populated clusters, the high ratio of singletons to clusters, and the large number of uncharacterized, conserved proteins confirms the need for more *Bacillus* phage isolation in order to understand the full extent of their diversity as well as their impact on host evolution.

## Background

Bacteriophages are the most abundant biological entities on the planet, with at least 10^31^ bacteriophages in Earth’s biosphere [1–5]. Their ability to infect and kill their bacterial hosts makes them key factors in both the evolution of bacteria and the maintenance of ecological balance (for recent reviews see [6–12]). In addition, they are able to infect and transfer genetic information to their hosts, in many cases being key factors in the transfer of pathogenic traits such as in pathogenic *Escherichia coli*, *Salmonella sp., Corynebacterium diphtheriae* and *Vibrio cholerae*. Despite their clear importance to global environmental and health concerns, little is known about the complexity and diversity of these living entities, but what is known from metagenomics and phage genome sequencing suggests it is vast.

The most studied bacteriophages are those that infect the Gram-positive bacterium *Mycobacterium smegmatis* mc^2^155, with over 4,800 phages isolated and 690 fully sequenced genomes (http://www.phagesdb.org). These phages have been isolated by students from throughout the world as part of the Howard Hughes Medical Institute Science Education Alliance Phage Hunters Advancing Genomics and Evolutionary Science (HHMI SEA-PHAGES) for determining the diversity of phages that can infect a single host. A recent analysis of 491 of these indicates they belong to approximately 17 “clusters” of related phages (A-Q) and 13 singleton clusters [13]. Of interest, identical mycobacteriophages have only been isolated independently twice (Graham Hatfull, personal communication). Beyond these *Mycobacterium* phages, the bacterial family with the most phages isolated infect the Gram-negative *Enterobacteriaceae* family (337 fully sequenced genomes available in GenBank). This group of phages has been isolated and sequenced independently from investigators throughout the world and contains many of the well-characterized, historical phages such as Lambda, Mu, T4 and T7. They have recently been grouped into 38 clusters of phages and 18 singleton clusters [14].

A third group of well-studied phages, the *Bacillus* phages, have also been isolated by diverse investigators from throughout the world and infect many strains of the genus *Bacillus*. The *Bacillus* genus is ubiquitous in nature and includes one of the best characterized model organisms, *B. subtilis,* as well as medically significant human pathogens, the most notorious being *B. anthracis* (the causative agent of anthrax) and *B. cereus* (which causes food poisoning). Phages have been isolated that infect *B. anthracis*, *B. cereus*, *B. megaterium, B. mycoides, B. pseudomycoides, B. subtilis, B. thuringiensis*, and *B. weihenstephanensis*, allowing a unique opportunity to investigate the diversity of phages that infect different hosts within a bacterial genus. This study focuses on the genomic comparison of 93 fully sequenced phages that infect the *Bacillus* genus and discusses their place in the diversity and evolution of these important bacteria. In addition, we identify several genes that may contribute to the pathogenicity of *Bacillus* species. This analysis presents a framework for understanding phages that infect *Bacillus* and for comparing *Bacillus* phage diversity with the diversity of phages that infect other genera. In addition, it increases our understanding of the evolution and diversity of phages and their hosts, including the evolution of pathogenic strains.

## Results and discussion

### Whole genome nucleotide and amino acid comparison of the *Bacillus*family of phages reveals 12 diverse clusters and 14 singletons

In order to determine the relationship of the 93 extant, fully-sequenced *Bacillus* phages as of June 1, we analyzed the published phage genomes by methods similar to those of Hatfull et al. [15, 16], including whole genome dot plot analysis, pairwise average nucleotide identities (ANI) and genomic maps. The accession numbers and basic properties (host, genome size, GC content, number of ORFs, number of tRNAs and morphotype) of the 93 full sequenced *Bacillus* phages are provided in Table 1 along with the appropriate reference.

**Table 1:** **Characteristics of reported** ***Bacillus*** **phages with complete genome sequences**

Cluster	Phage name	Host	Size (bp)	GC%	ORFS	tRNA	Accession Number	Family	Ref.
A1	Wip1	A	14319	36.84	27	0	NC_022094	*T*	[17]
A1	AP50	A	14398	38.65	31	0	NC_011523	*T*	[18]
A2	GIL16c	T	14844	39.72	32	0	NC_006945	*T*	[19]
A2	Bam35c	T	14935	40.08	31	0	AY257527	*T*	[19]
A2	pGIL01	T	14931	39.73	30	0	AJ536073	*T*	[20]
B1	Phi29	S	19282	39.99	27	0	EU771092.1	*P*	[21]
B1	PZA	S	19366	39.66	27	0	M11813	*P*	[22]
B2	B103	S	18630	37.66	17	0	NC_004165	*P*	[23]
B2	Nf	S	18753	37.32	27	0	EU622808	*P*	
B3	Gir1	B	21129	34.65	34	0*		*P*	***
B3	GA-1	B	21129	34.66	35	1	X96987	*P*	[24]
C1	MG-B1	W	27190	30.75	42	0	NC_021336	*S*	[25]
C2	Stitch	B	24320	30.36	37	0*		*P*	***
D1	Page	M	39874	40.71	50	0*	NC_022764		[26]
D1	Poppyseed	M	39874	40.71	50	0	KF669657		***
D1	Pony	M	39844	40.70	48	0	NC_022770	*P*	[27]
E1	TP21-L	C	37456	37.80	56	0	NC_011645	*S*	[28]
E1	BMBtp2	T	36932	37.79	53	0	NC_019912	*S*	***
E1	ProCM3	T	43278	37.36	66	0*	KF296717	*S*	***
F1	γ isolate d'Herelle	A	37373	35.13	53	0	DQ289556	*S*	[29]
F1	γ isolate 51	A	37253	35.22	53	0	DQ222853	*S*	***
F1	WBeta	A	40867	35.26	53	0	DQ289555	*S*	[29]
F1	Gamma	A	37253	35.22	53	0	NC_007458	*S*	[30]
F1	Cherry	A	36615	35.27	51	0	DQ222851	*S*	27)
F1	γ isolate 53	A	38067	35.10	50	0	DQ222855	*S*	
F1	Fah	B	37974	34.95	50	0	NC_007814	*S*	[31]
F2	phiCM3	T	38772	35.48	56	0*	NC_023599		***
F2	phIS3501	T	44401	34.86	51	1	JQ062992	*S*	***
F2	BtCS33	T	41992	35.22	57	0	NC_018085	*S*	[32]
F3	BceA1	C	42932	35.66	63	0	HE614282		[33]
G1	IEBH	T	53104	36.42	86	0	EU874396	*S*	
G1	250	C	56505	36.44	54	0	GU229986		[34]
H1	Andromeda	P	49259	41.91	79	0	NC_020478	*S*	
H1	Gemini	P	49362	41.9	79	0	KC330681	*S*	
H1	Glittering	P	49246	42.05	78	0	NC_022766	*S*	[35]
H1	Curly	P	49425	41.82	77	0	NC_020479	*S*	
H1	Eoghan	P	49458	42.21	75	0	NC_020477	*S*	
H1	Taylor	P	49492	42.29	75	0	KC330682	*S*	
H1	Riggi	P	49836	41.46	79	0	NC_022765	*S*	[36]
H1	Blastoid	P	50354	42.23	79	0	NC_022773	*S*	[37]
H1	Finn	M	50161	41.69	77	0	NC_020480	*S*	
H1	Polaris	B	33403	42.61	45	0*			***
I1	Pleiades	B	64698	47.66	112	0*			***
I1	Pappano	B	65662	47.57	113	0*			***
J1	Staley	M	81656	35.35	113	0	NC_022767	*S*	[38]
J1	Slash	M	80382	35.23	111	0	KF669661	*S*	[39]
J2	Basilisk	C	81790	33.9	141	2	KC595511	*S*	[40]
K1	SPO1	S	132562	39.97	204	5	NC_011421	*M*	[41]
K1	Pegasus	B	146685	40.3	236	3*			[42]
K1	CampHawk	S	146193	40.2	231	2	NC_022761	*M*	[43]
K2	Shanette	C	138877	40.8	223	3	KC595513	*M*	[40]
K2	JL	C	137918	40.8	222	4	KC595512	*M*	[40]
L1	phiNIT1	P	155631	42.12	219	4	NC_021856		
L1	Grass	S	156648	42.25	252	3	NC_022771		[44]
L2	SI0phi**	S	146698	39.02	206	0*	KC699836	*M*	
L3	phiAGATE	P	149844	49.97	210	4	NC_020081		[45]
L4	Bastille	C	153962	38.14	280	7	JF966203	*M*	[45]
L4	Evoli	T	159656	38.06	293	8	KJ489398		
L4	HoodyT	T	159837	38.01	299	8	KJ489400	*M*	
L4	CAM003	T	160541	38.03	296	8	KJ489397		
L4	JPB9	B	159478	38.00	322	5*			***
L5	B4	C	162596	37.71	277	0	JN790865		
L5	Troll	T	163019	37.83	289	0	NC_022088	*M*	
L5	Spock	T	164297	37.62	283	0	NC_022763	*M*	
L5	Adelynn	B	165049	37.77	293	0*			[46]
L5	BigBertha	T	165238	37.77	291	0	NC_022769	*M*	[47]
L5	Riley	B	162816	37.78	290	0	NC_024788.		***
L5	B5S	C	162598	37.71	272	0	JN797796	*M*	[48]
L6	BCP78	C	156176	39.86	227	18	JN797797	*M*	[49]
L6	BCU4	C	154371	39.86	223	19	JN797798	*M*	[50]
L7	BCP1	C	152778	39.76	227	17*	KJ451625	*M*	
L7	Bc431v3	C	158621	39.98	238	21	JX094431	*M*	[51]
L8	Hakuna	T	158100	38.70	294	0	KJ489399H		
L8	Doofenshmirtz	B	161793	38.74	294	0*			***
L8	Nagalana	B	163041	38.75	302	0*		*M*	***
L8	Megatron	T	158750	38.80	291	0	KJ4894011H		
L8	BPS10C	C	159590	38.74	271	0	NC_023501	*M*	[52]
L8	BPS13	C	158305	38.75	268	0	JN654439	*M*	[52]
L8	W. Ph.	C	156897	36.45	274	0	HM144387	*M*	[26]
Single	BV1	B	35055	44.85	54	0	DQ840344		
Single	phBC6A52	C	38472	34.72	49	0	NC_004821		
Single	phi105	M	39325	42.69	51	0	NC_004167	*S*	[53]
Single	BCJA1C	B	41092	41.74	58	0	NC_006557	*S*	
Single	PBC1	C	41164	41.68	50	0	JQ619704	*S*	[54]
Single	SPP1	M	44010	43.72	99	0	NC_004166	*S*	[55]
Single	PM1	S	50861	41.29	86	0	NC_020883	*S*	[56]
Single	phBC6A51	T	61395	37.69	75	0	NC_004820		[57]
Single	BCD7	C	93839	38.04	140	0	JN712910		
Single	SPBc2	S	134416	34.64	185	0	NC_001884	*S*	[58]
Single	SP10	S	143986	40.49	236	0	NC_019487	*M*	[40]
Single	BanS-Tsamsa	A	168876	34.32	272	19	NC_023007	*S*	[59]
Single	0305phi8-36	T	218948	41.8	246	0	NC_009760	*M*	[59]
Single	G	M	497513	29.93	675	18*	JN638751	*M*	[60]

Hosts are the bacterial hosts on which the phages were isolated (not the host range) and are abbreviated as *Bacillus anthracis* (A), *Bacillus cereus* (C), *Bacillus sp.* (B), *Bacillus megaterium* (M), *Bacillus pumulis* (P), *Bacillus subtilis* (S), *Bacillus thuringiensis* (T), and *Bacillus weihenstephanensis* (W). ORFs are the number of Open Reading Frames predicted to be encoded by the genome as provided in the reported annotation. Family is *Myoviridae* (M), *Siphoviridae* (S) or *Podoviridae* (P). A reference (Ref.) for the published genome is provided when available.

*tRNA predicted in this study using Aragorn and DNAMaster.

**Phage SI0phi is reported as an incomplete genome but is included in this analysis because it was complete enough to clearly assign it to a cluster.

***Indicates phage sequences obtained through phagesdb.org.

**Figure 1:**
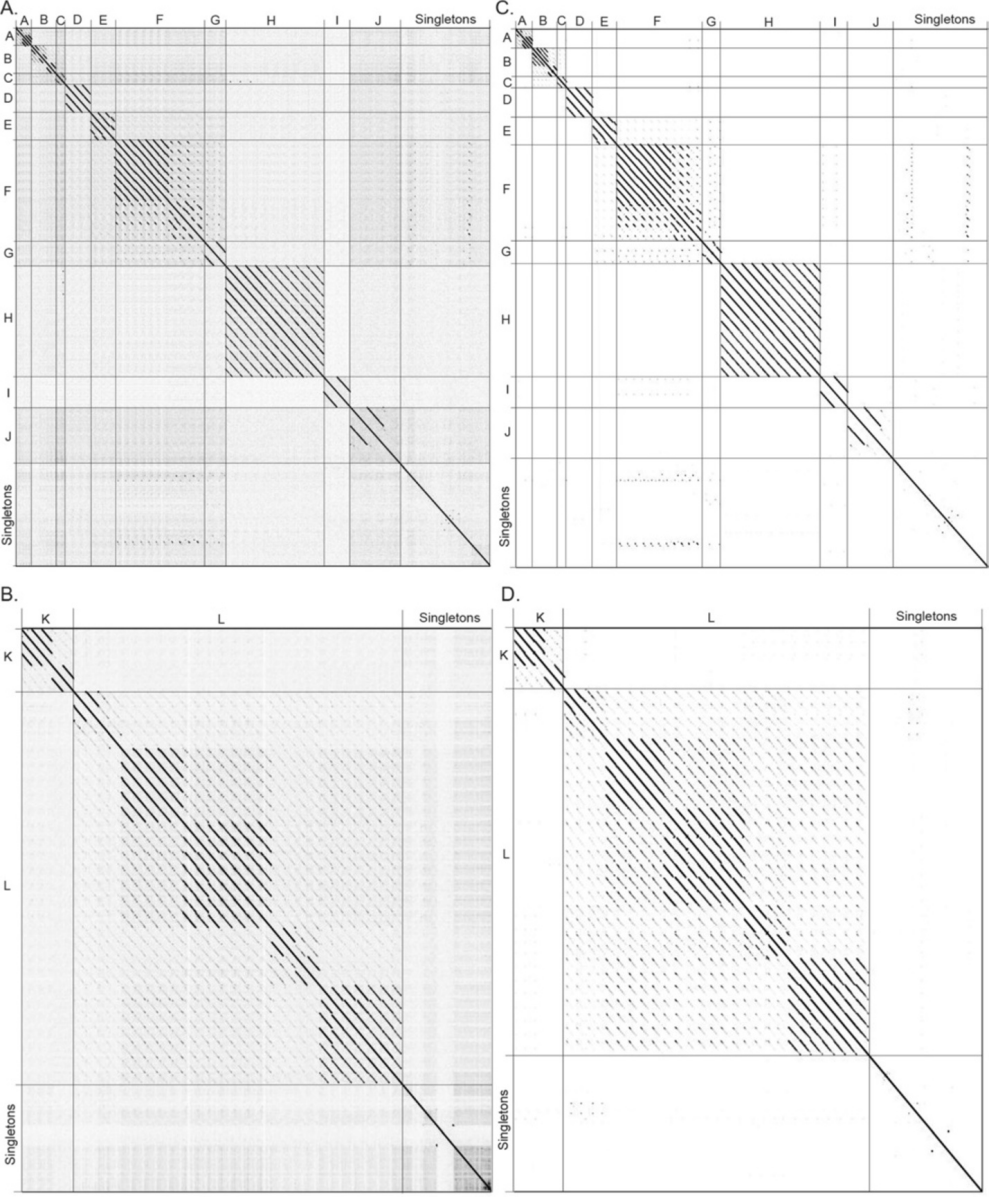
**Nucleotide and amino acid dot plot analysis of 93 fully sequenced** ***Bacillus*** **phages reveals 12 clusters (A-L) and 14 singletons.** Nucleotide **(A)** and amino acid **(C)** dot plot of *Bacillus* genomes of less than 100 kb organized by similarity reveals 10 clusters of related phages. Nucleotide **(B)** and amino acid **(D)** dot plot of *Bacillus* genomes of greater than 100 kb organized by similarity reveals 2 clusters of related phages. Thick lines indicate cluster assignments, which are provided on the Y-axis (A-L). Dot plots were produced using Gepard [61] and whole genome amino acid sequences were retrieved from Phamerator [62].

Dot plot analysis of the *Bacillus* phages revealed 12 clusters of phages with similarity over at least 50% of their genomes (clusters A through L) and 14 phages that are singletons, having little to no nucleotide similarity to any other *Bacillus* phages. Genomic dot plot analysis consists of placing the nucleotide sequences across both the X- and Y-axis. A dot is placed where the sequences are identical resulting in a diagonal line down the center of the plot when a sequence is compared to itself. The phages were aligned on two separate plots due to the wide range in genome size and the fact that no additional nucleotide similarity was seen in a combined plot. Figure 1A contains phage genomes less than 100 kb while 1C contains the larger phage genomes. As stated above, assignment of a phage to a cluster was based on nucleotide similarity over at least 50% of the genome when compared to at least one other phage in the cluster. Thus, a phage could be placed into the same cluster by weak similarity over most of the genome, by strong similarity over about half of the genome, or by a combination of relatedness. The ANI values were also calculated within each cluster and found to be at least 55% between a phage and another phage within a cluster. From the total of 26 clusters just over half (14) are singleton clusters containing a single phage member, suggesting that the isolation of unique *Bacillus* phages is far from complete.

In addition to showing strong evolutionary relationships, whole genome nucleotide dot plots also reveal smaller regions of homology (<50% span length) between phages of different clusters that are likely areas of recombination. The largest such region is a ~10,000 bp region of similarity between phBC6A51 (bp 44289–50616 and 58088–61389) and cluster F phages that includes a tail component protein, minor structural protein and holin as well as a site-specific recombinase, a Ftsk/SpoIIIE family protein and five conserved phage proteins.

In addition to whole genome nucleotide analysis, whole proteome dot plot analysis was performed (Figures 1B and D). Because nucleotide sequences diverge more rapidly, the amino acid dot plots were expected to reveal more distant evolutionary relationships. The analysis confirmed the basic cluster assignments seen with whole genome nucleotide analysis and revealed distant relationships between the E, F, G and I clusters discussed in more detail below. Note that there should be some limited similarity between all of the *Bacillus* tailed phages in that they should all encode a major capsid protein (MCP), portal protein and terminase. However, these proteins can diverge to a point that no sequence similarity is apparent.

Another common way to group phages is by the percent of the proteome that is conserved between phages. CoreGenes 3.0 was used to confirm clusters by ensuring that phages within a cluster share ~40% of their proteome, a cutoff commonly used for determining phage relationships [63, 64]. The cluster with the lowest conservation of the proteome (that is, the lowest conservation between a phage and its closest relative) is the F cluster, with the highly related phages Staley and Slash sharing only 43.4% of their proteome with Basilisk. All other clusters yielded proteome comparison scores well above the 40% CoreGenes threshold, confirming that the phages belong in the proposed clusters.

The division of phages into the proposed clusters is also supported by the low standard deviation in the average basic phage properties including genome size, GC content, number of ORFs and morphotype (Table 2). For example, cluster A consists completely of tectiviruses of an average genome size of 14685 ± 302 bp, clusters B and D of podoviruses with short tails (average genome size is 19715 ± 1132 and 39864 ± 17 bp, respectively), clusters C, E, F, G, H and J of long noncontractile siphoviruses (average genome size ranging from 25755 ± 2029 to 81276 ± 777 bp), and the large contractile myovirus clusters K and L (average genome size is 140447 ± 5978 and 158753 ± 4550 bp, respectively). Cluster I is of unknown morphotype. The average number of tRNA’s for each cluster is also reported but is highly variable within a cluster with standard deviations often approaching the number of tRNA’s. This variation may reflect the phages’ adaptation to different hosts since tRNA’s are thought to provide efficient protein production in hosts with alternate codon preferences [65]. Further host range studies are needed to test these hypotheses.

**Table 2:** **Summary of** ***Bacillus*** **cluster phage characteristics**

Cluster	Sub.	Phages	Hosts	Genome size	%GC	# ORFS (tRNA)	Type
A	2	5	A, T	14685 ± 302	39.0 ± 1,3	30.2 ± 1.9(0)	T
B	3	6	B, S	19715 ± 1132	37.3 ± 2.3	27.8 ± 6.5(0.2 ± 0.4)	P
C	2	2	B, W	25755 ± 2029	30.6 ± 0.3	39.5 ± 3.5(0)	S
D	1	3	M	39864 ± 17	40.7 ± 0.0	49.3 ± 1.2(0)	P
E	1	3	C, T	39222 ± 3522	37.7 ± 0.3	48.7 ± 10.2(0)	S
F	3	11	A, B, C, T	39409 ± 2677	35.2 ± 0.2	53.6 ± 3.8(0.1 ± 0.3)	S
G	1	2	C, T	54805 ± 2405	36.4 ± 0.0	70.0 ± 22.6(0)	S
H	1	10	B, M, P	48000 ± 5143	42.0 ± 0.3	74.3 ± 10.4(0)	S
I	1	2	B	65180 ± 682	47.6 ± 0.1	112.5 ± 0.7(0)	UK
J	2	3	C, M	81276 ± 777	34.8 ± 0.8	122 ± 16.8(0.7 ± 1.2)	S
K	2	5	B, C, S	140447 ± 5978	40.4 ± 0.4	223 ± 12.2(3.4 ± 1.1)	M
L	8	27	B, C, P, S, T	158753 ± 4550	39.1 ± 2.5	269.7 ± 32.2(5.0 ± 6.7)	M

Characteristics given are cluster assignment, number of subclusters (Sub.), number of phages in the cluster, host species from which the phages were isolated, the average genome size, average percent GC content, average number of ORFS with average number tRNA in parenthesis, and the morphotype. Averages are given with the standard deviation. Species abbreviations are *Bacillus anthracis* (A), *Bacillus cereus* (C), *Bacillus sp.* (B), *Bacillus megaterium* (M), *Bacillus pumulis* (P), *Bacillus subtilis* (S), *Bacillus thuringiensis* (T), and *Bacillus westenstephanensis* MG1, (W). Family/morphotype abbreviations are *Tectiviridae* (T), *Podoviridae* (P), *Siphoviridae* (S), and *Myoviridae* (M). UK is unknown/unreported.

### Division of clusters into subclusters reveals large variance between clusters

**Figure 2:**
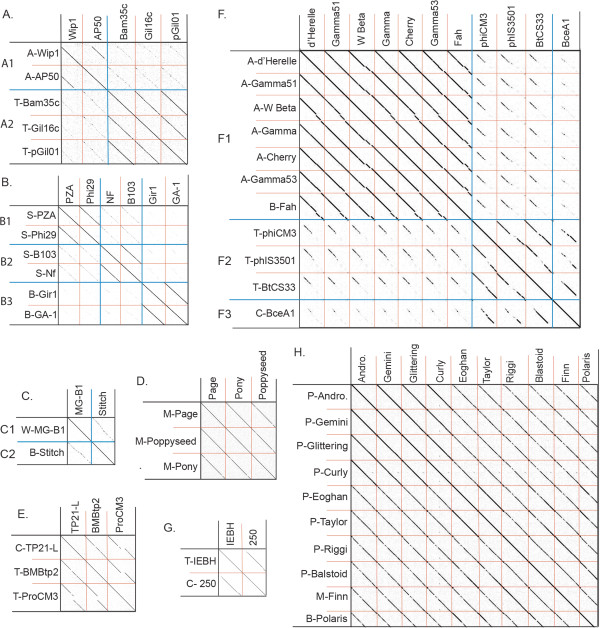
**Analysis of fully sequenced** ***Bacillus*** **phage genomes belonging to clusters A through H reveals 15 subclusters. (A-H)** Bacillus phage clusters A through H, respectively. Subcluster divisions are provided by blue lines and are indicated on the Y-axis when there are more than one per cluster. Individual phages are separated by red lines. Phage names are provided on the X-axis and Y-axis with host abbreviation from which the phages were isolated were isolated indicated on the Y-axis. Hosts abbreviations are *Bacillus anthracis* (A), *Bacillus cereus* (C), *Bacillus sp.* (B), *Bacillus megaterium* (M), *Bacillus pumulis* (P), *Bacillus subtilis* (S), *Bacillus thuringiensis* (T), and *Bacillus weihenstephanensis* MG1, (W). Dot plots were produced using Gepard [61]. Phage Andromeda is abbreviated (Andro.).

**Figure 3:**
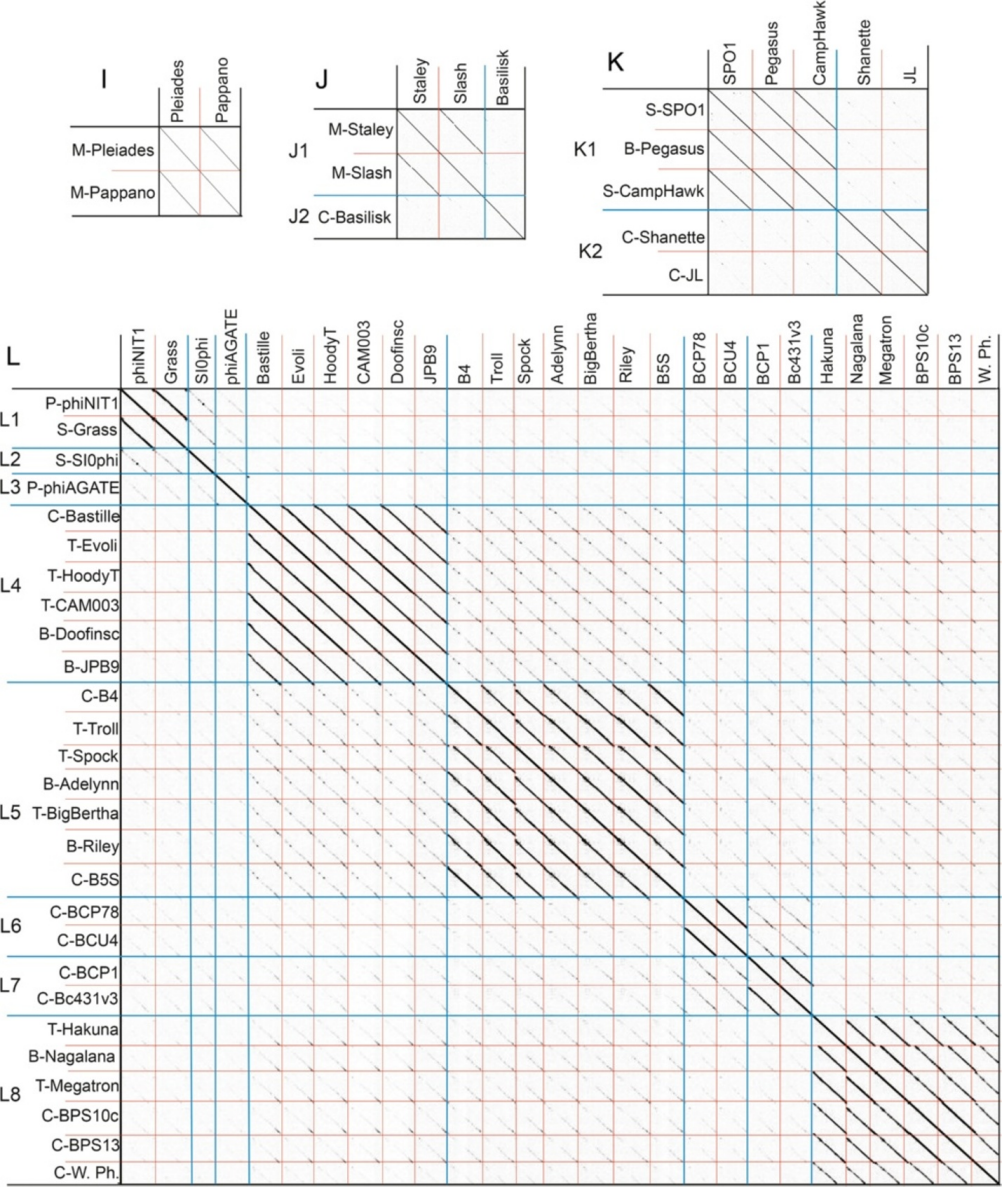
**Analysis of fully sequenced** ***Bacillus*** **phage genomes belonging to clusters I through L reveals 13 subclusters. (I-L)** Bacillus phage clusters I through L, respectively. Subcluster divisions are provided by blue lines and are indicated on the Y-axis when there are more than one per cluster. Individual phages are separated by red lines. Phage names are provided on the X-axis and Y-axis with host abbreviation from which the phages were isolated indicated on the Y-axis. Hosts abbreviations are *Bacillus anthracis* (A), *Bacillus cereus* (C), *Bacillus sp.* (B), *Bacillus megaterium* (M), *Bacillus pumulis* (P), *Bacillus subtilis* (S), *Bacillus thuringiensis* (T), and *Bacillus weihenstephanensis* MG1, (W). Dot plots were produced using Gepard [61].

Each cluster was further analyzed by nucleotide dot plot to reveal groups of high similarity, or subclusters (Figures 2 and 3). These subclusters were chosen based on natural divisions in phage similarity seen in the dot plot, but could be more strictly defined by ANI values of at least 66% between two phages within the subcluster. The subcluster assignments indicate great diversity in the relatedness within each *Bacillus* phage cluster. It is unknown whether this diversity represents evolutionary forces that constrain certain types of phages or if it is an artifact of phage isolation. Further phage isolation is necessary for this distinction.

#### Clusters containing highly related phages

Clusters C, D, E, H, and I are each comprised of a single subcluster containing highly related phages (sharing at least 74% ANI). Cluster H is the largest cluster and contains 10 highly related siphovirus phages, the cluster D and cluster E each contain three phages of the podovirus and siphovirus families, respectively, cluster C contains two siphoviruses, and cluster I contains two phages of unknown morphotype. The majority of phages in each of these clusters are recently isolated phages that are not well-characterized. In fact, the MCP was not annotated for any cluster D, E, H or I phage and we were unable to identify an MCP by TBLASTN searches, suggesting that the MCP of these phages are novel.

#### Clusters containing more distantly related phages

Clusters A, B, F, G, J, K and L all contain multiple subclusters, with B, F, J and K being the most variable. Cluster B contains three subclusters having ANI values ranging from 48% to 76% between phages (but all phages have at least 54% with at least one other phage in a different subcluster). A CoreGenes 3.0 analysis confirms this relationship of cluster B phages, with B1 phages sharing 96% of their proteome within the subcluster but approximately 63% and 56% with the B2 and B3 cluster proteomes, respectively. Similarly, cluster F contains 11 phages divided into 3 subclusters where ANI varies from 42% to 99.99% between phages but all phages have at least 55% to one another. There is 86% proteome conservation within each subcluster, and between subclusters there is at least 41% proteome conservation. Cluster J harbors the very similar Staley and Slash (94% ANI) and the more distantly related phage Basilisk, which shares ~55% ANI and 43% of its proteome with Staley/Slash. Cluster K harbors SPO1 and close relatives CampHawk and Pegasus (subcluster K1) as well as the more distantly related phages Shanette and JL (subcluster K2), which share ~53% of their proteomes with the K1 phages.

Clusters G and L contain more closely related phages. Cluster G harbors siphoviruses IEBH and 250 which share 90% ANI and 55% of their proteomes. L is the largest cluster and contains 27 phages that are likely to all be myoviruses since 15 are reported as such. Of interest, these seven subclusters to which these large phages belong are highly variable in host, tRNA content and number of ORF’s (see Table 1), but they are all highly related having at least 81% ANI.

Overall, *Bacillus* phages remain highly uncharacterized but clusters F and K contain a couple of well-characterized *Bacillus* phages including the *B. anthracis* typing phages Gamma and Cherry and *B. subtilis* phages SPO1 and CampHawk, respectively.

### Single gene product analysis mirrors whole genome/proteome analysis

**Figure 4:**
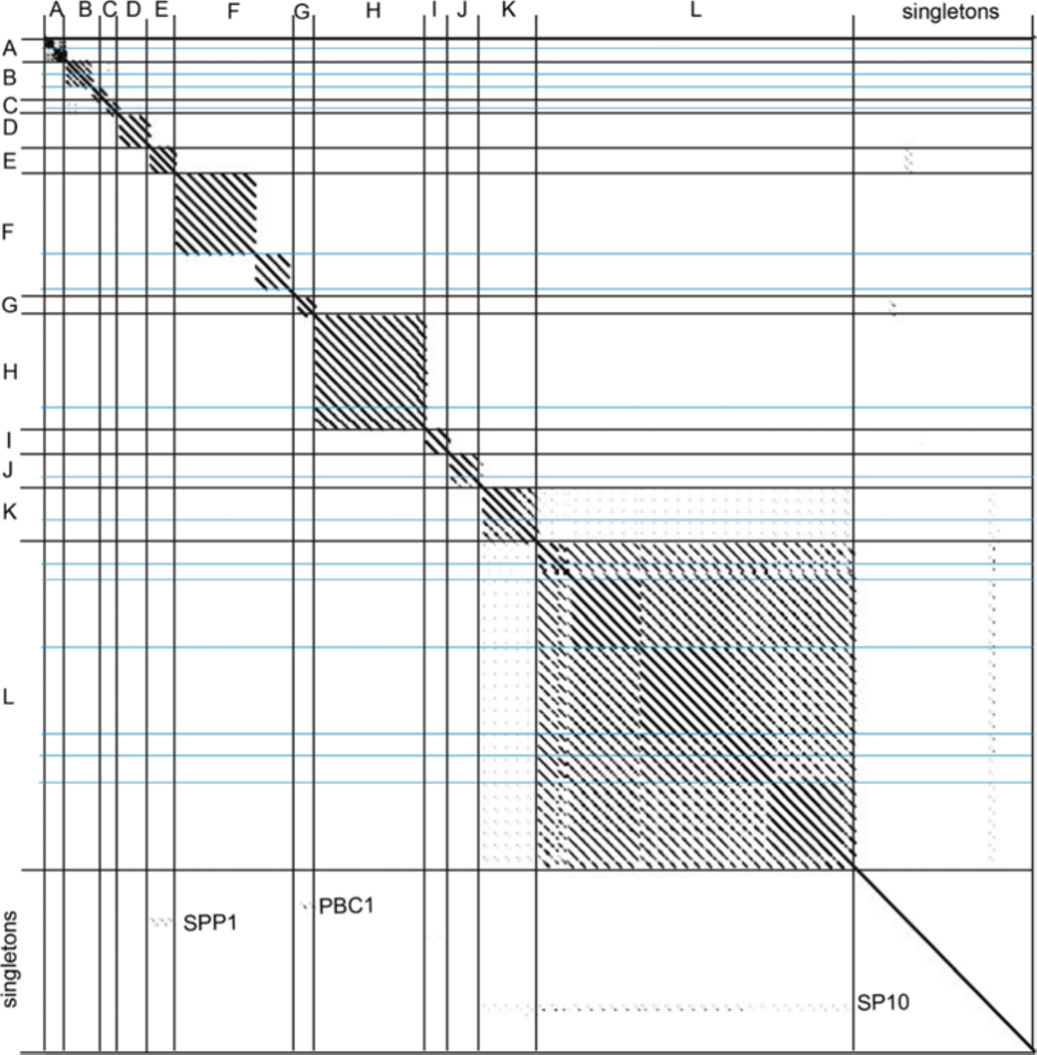
**Single gene amino acid dot plot analysis using the large terminase mirrors whole genome cluster assignment of** ***Bacillus*** **phages.** *Bacillus* phage clusters A-L are indicated on both the X-and Y-axis. Sequences for comparison were chosen by annotated large terminase gene products or a BlastP alignment to the closest relative when unannotated. Dot plots were produced using Gepard [61].

**Figure 5:**
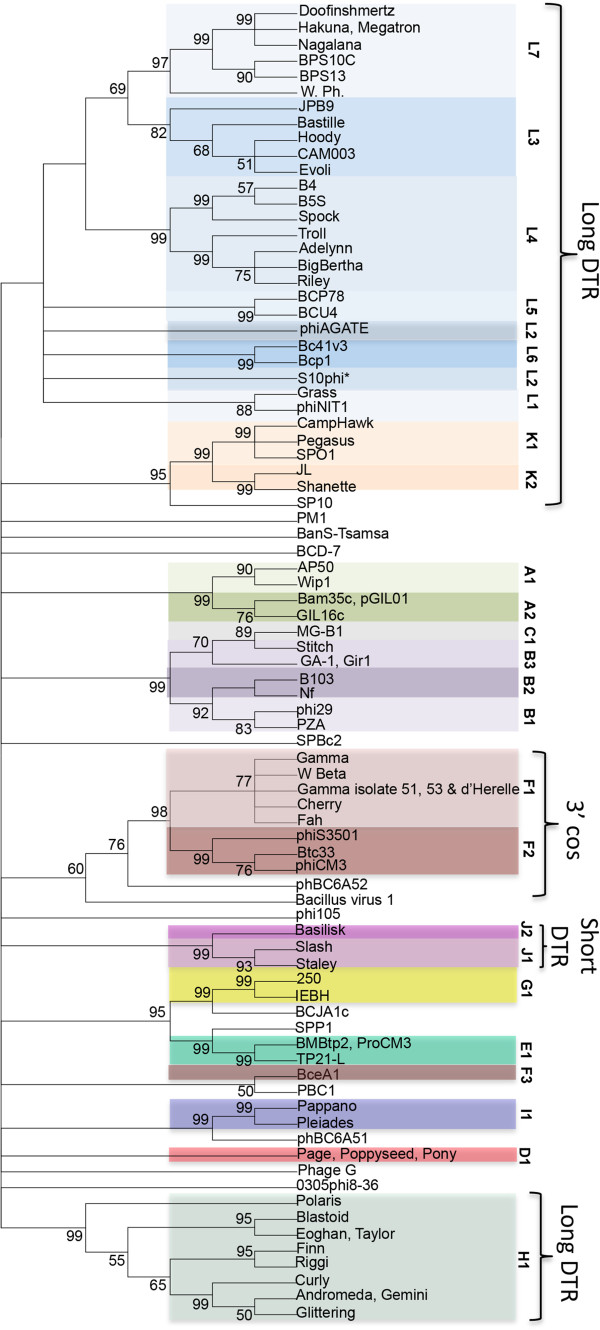
**A neighbor-joining tree analysis of the** ***Bacillus*** **terminase mirrors whole genome cluster assignments.** Phage names are colored by whole genome subcluster assignment and this subcluster assignment is indicated on the right. Putative replication strategies for phages are also indicated when known. Abbreviations are direct, terminal repeats (DTR) and cohesive ends (cos). The phylogenetic tree was constructed using a MUSCLE [69] alignment and the neighbor-joining method in Mega5 [70]. Bootstrapping was set to 2000 and the unrooted tree was collapsed at a less than 50% bootstrap value.

In addition to using whole genome or proteome comparisons to determine phage cluster assignment we recently demonstrated the utility of single gene product analysis using the mycobacteriophage tape measure protein (TMP) and major capsid protein (MCP) gene products [66] *. W*e were unable to use either TMP or MCP for *Bacillus* phage single-gene comparison because podoviruses do not have a TMP and the MCP was not reported or identified by a TBLASTN search for 18 of the 93 *Bacillus* phages (including clusters D, E, H and I). Three genes are thought to be common to all tailed phages, the MCP (the major constituent of the icosahedral shell), portal protein (forms the pore into the capsid through which the DNA is packaged) and large terminase (the ATPase that packages the DNA into capsid) [67]. A putative large terminase gene product (TerL) was identified in 100% of the *Bacillus* phages and was, therefore, used for single-gene comparison (Figure 4). A dot plot alignment of the terminase gene products (TerL) confirmed our basic cluster/subcluster assignment with 100% of phages grouping by their pre-assigned clusters or subclusters, and 11 of 14 singletons remaining singletons. This overall percentage (96.8%) is comparable to the 98.8% reported for the mycobacteriophages using TMP [66] *.* The terminase dot plot analysis is supported by a neighbor-joining tree in which all of the proteins grouped by cluster/subcluster and the same three singletons were associated with another cluster (Figure 5). The few outliers are consistent with a recent analysis that suggested genes encoding TerL have undergone sufficient horizontal transfer between phage groups to disrupt some correlations between terminase sequence type and cluster relationship [68].

From single-gene comparison, two of the subclusters appear to be unrelated to the rest of the cluster in which they belong (subcluster B3 and F3) while three singletons (SPP1, PBC1 and SP10) display remarkable similarity to the D, F/G or K/L clusters, respectively, as seen by both dot plot and neighbor-joining tree analysis. These relationships could indicate more distant/ancient relationships over the entire chromosome or small regions of genetic exchange. The limited similarity of subcluster B3 and F3 TerL proteins to the rest of the B and F clusters is consistent with their distant whole genome/proteome relationships (faint diagonal lines on both the nucleotide and amino acid dot plots, see Figure 1). In contrast, CoreGenes analysis suggests small regions of genetic exchange for SSP1 in that it shares only ~5% of its proteome with the cluster D phages (including the terminase, tailspike, DnaB/DnaD replication protein, and the single stranded DNA binding and annealing proteins).

### Predicting phage replication strategies by terminase conservation

The identification and analysis of *Bacillus* phage terminase proteins presented in Figure 5 can also provide valuable insight into the replication strategy of these highly uncharacterized phages by comparing their terminases to those of well-characterized phages. Such comparisons have been used to determine the replication strategy of phages that infect *Enterobacteriaceae* hosts as well as phages that infect *Paenibacillus larvae* [71, 72]. In our analysis, several *Bacillus* phages contain terminases that were similar to the well-characterized SPO1 *Bacillus* phage, suggesting that they replicate and package their DNA by a similar concatemer strategy resulting in non-permuted DNA with long, direct terminal repeats [73, 74]. The cluster K phages had terminases of at least 87% similarity to SPO1 by BLASTP, while clusters H and L were weakly similar (~43% and ~56% similar respectively) and singleton phage SP10 was 68% similar. Cluster F, phBC6A52 and Bacillus virus 1 terminases have weak homology to the HK97 terminase (42%- 45% similarity) which packages by 3’ cos ends, while phages of cluster J and singleton BanS-Tsamsa may have short DTRs due to weak homology to the *Clostridium* phage C terminase (~47% similarity) [75].

### Identification of two superclusters describing distantly related phages through proteome conservation analysis

**Figure 6:**
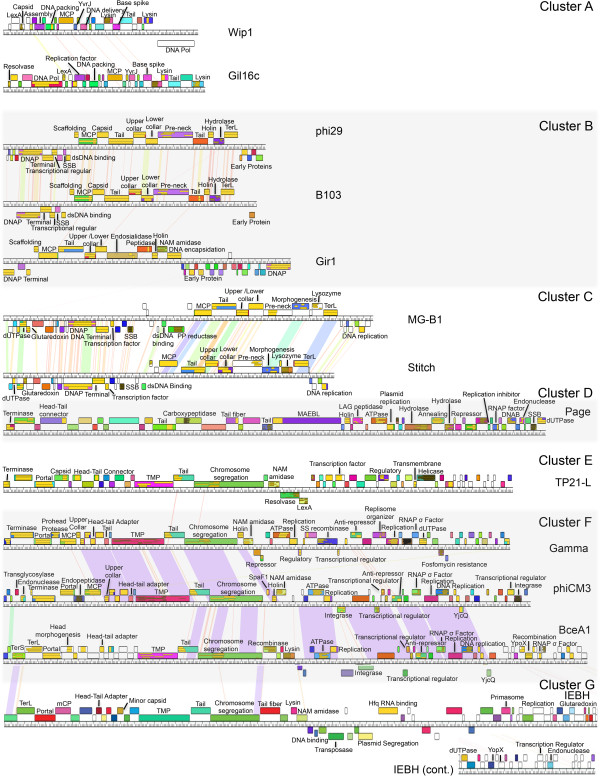
**A comparison of gene content and order within the** ***Bacillus*** **phage clusters reveals modularity and great diversity.** Genome maps for representative phages from the subclusters within Bacillus phage clusters A-G are provided. Phages were mapped using Phamerator [62], where purple lines between phages denote regions of high nucleotide similarity and the ruler corresponds to genome base pairs. Boxes for gene products are labeled with predicted function, occasionally numbered, and colored to indicate similarity between the phages (E-value <1e^−4^). Abbreviations are adenosine triphosphatase (ATPase), DnaB helicase (DNAB), double-stranded DNA binding (dsDNA binding), 2'-deoxyuridine 5'-triphosphatase (dUTPase), major capsid protein (MCP), N-acetyl-muramyl-L-alanine amidase (NAM amidase, pyrophosphate reductase (PP reductase) RNA polymerase (RNAP), sigma factor (σ factor), large terminase (TerL), small terminase (TerS), tape measure protein (TMP), pilus specific protein, ancillary protein involved in adhesion (SpaF1), single-stranded binding protein (SSB), single-strand recombinase (SS recombinase).

In an effort to identify more distantly related phages belonging to “superclusters”, we carefully analyzed faint nucleotide and proteome dot plot lines, CoreGenes percentages, and whole genome maps for intercluster relationships. The genomic map of a representative phage from each subcluster is given in Figure 6 as an example, however the larger phages are excluded due to space constraints (clusters A through G are shown). Because short regions of similarity are common among phages, phages had to have similarity in genome content and order (synteny) to be termed a supercluster. Table 3 lists the two superclusters identified in this analysis.

Faint lines can be seen in both the nucleotide and proteome dot plots between clusters E, F and G as well as singleton PBC1. In addition, a similar genome content and order can be seen between these phages (for example phages TP21-L, Gamma and IEBH) where the first section of the chromosome contains phage structure and assembly genes and the last section harbors DNA metabolism genes (see Figure 6). These clusters also share an appreciable percentage of their proteome, with cluster E, F and G phages sharing ~21% of their proteome with at least two members of another cluster. This observation suggests an ancient relationship that has diverged. Singleton PBC1 also shares 32% of its proteome with the cluster G phages. These proteins include the portal protein, the MCP, three putative minor capsid proteins, a putative minor structural protein, the TMP, a holin, a glutaredoxin-like protein and nine hypothetical proteins. We have termed this supercluster the d’Herelle-like supercluster after the founding phage.

**Table 3:** ***Bacillus*** **phage superclusters describe distantly related phages sharing significant proteome conservation**

Supercluster	Phages	% Proteome conserved*
d’Herelle-like	Clusters E, F and G, phage PBC1	21% (E, F and G), 32% (G and PBC1)
SPO1-like	Clusters K, Cluster L	27%

*Percent proteome conserved is the percentage conserved between two phages within different cluster as determined by CoreGenes.

Clusters K, L and singleton SP10 have similar relationships, with K and L cluster phages sharing up to 27% of their proteome. Singleton SP10 shares ~29% of its proteome with cluster K phages and ~24% with cluster L phages, including several structural proteins (portal protein, MCP, minor structural protein, tail sheath, tail tube, tail assembly chaperone, tail lysin, tail fiber, tail baseplate and tail spike proteins), DNA replication proteins (DNA helicases, primase, endonuclease, exonuclease, and ribonuclotide reducatase), a peptidoglycan binding protein, a tRNA processing protein, several RNA polymerase sigma factors, and hypothetical proteins. Of interest, phage SP10 had previously been described as a SPO1-related phage by its discoverers [76]. This supercluster comprised of clusters K, L and singleton SP10 is termed the SPO1-like supercluster after this well-characterized *B. subtilis* phage.

Although faint lines can also be seen between the B and C clusters in some dot plots and 29% of the proteome is conserved between phages of this cluster, whole genome map displays different genome content and order (see Figure 6). In this case, rather than the phages displaying some similarity over a majority of the genomic map, they displayed similarity over a small portion. These phages were not included in a supercluster due to the very limited similarity as well as the differences in gene synteny, which suggest differences in phage lifestyles. These results reinforce the need for several analytical approaches in determining phage relationships.

### DNA metabolism, cell lysis, structural, and host gene products are well-conserved in *Bacillus*phages

Phamerator [62] was used to determine the most highly conserved gene products within the 93 fully sequenced *Bacillus* phages, and the extent of conservation among the phages. Phamerator identified a total of 4,922 phams, or groups of proteins with homology to one another. Of these, 951 (19%) had a predicted function and 3,971 (81%) were uncharacterized. In addition, 3,058 (62%) were orphams (phams containing a gene product from a single phage). This analysis confirms the highly diverse and uncharacterized nature of the *Bacillus* phages and underscores the immense biological reservoir that is present. Table 4 (phams with predicted function) and Table 5 (phams with uncharacterized proteins) contain the highly conserved phams that have over 20 members. These phams are partitioned by their function as DNA replication/metabolism proteins, virion structure and assembly proteins, cell lysis proteins, or proteins involved in host function. It is important to note that there may be other proteins with similar function not included in a pham due to lack of homology.

**Table 4:** **Common** ***Bacillus*** **phage proteins of predicted function with over 20 members**

Pham #*	Domain/function	# Members	# Phages	Phages (cluster or phage name)
**Dna Replication/metabolism**				
236	DNA Polymerase	52	33	K,L1,L2,L3,L4,L5,L6,L8,SP10,Bc431v3
247	Ribonucleotide reductase	47	41	I,J,K1,L,0305φ8-36,L6,L3,BCD7,SP10,BanSTsamsa,SPB2
261	Helicase	36	33	L,K,SP10
256	Exonuclease	34	33	L,K,SP10
257	Nuclease	33	32	K,L1,L2,L3,L4,L5,L6,L7BPS10C,SP10,W.PH.,
101	Dihydrofolate Reducatase	32	31	Hakuna,Nagalana,Megatron G,J2,L,BCD7,BanS-Tsamsa
98	Thymidylate Synthase	30	28	L,BCD7
99	dNTP MonoP Kinase	30	29	I,L
246	Ribonucleotide diP Reduct.	29	27	J1,K1,L4,L5,L6,L8,0305phi8-36
232	DNA Polymerase III	29	27	L
238	Histone	28	27	L
252	dU Nucleotidylhydrolase	28	27	L
258	Replicative DNA Helicase	28	27	L
229	RecA	28	27	L
254	DNA Primase	28	27	L
227	Sigma Factor	28	27	L
244	Glutaredoxin	25	24	L1,L2,L4,L5,L6,SPBc2,BPS10c,BPS13,
370	DNA Segregation ATPase	24	24	Megatron,Nagalana,W.Ph
740	Met S-methyltransferase	24	16	E,F1,Andromeda,Gemini,Glittering,Curly,Eoghan,Taylor, Riggi,Blastoid,Finn,BV1,PL1,E1,phIS3501,phBC6A51 L5,L8,Bastille,Doofenshmirtz,JPB9,W.Ph
**Virion structure and assembly**				
278	Tail Assembly Chaperone	56	27	L
274	Tail Fiber	43	29	J2,L ,BanS-Tsamsa
5174	Phage Terminase	41	33	K,L,SP10
264	Adsorption Tail	36	33	L1,L2,L3,L5,L6,L8,Bc431v3
295	Portal Protein	35	33	K,L,SP10
291	MCP	35	33	K,L,SP10
276	Tail Lysin	35	33	K,L,SP10
266	Baseplate	34	33	K,L,SP10
283	Structural Protein	34	33	K,L,SP10
284	Tail Sheath	34	33	K,L,SP10
293	Prohead Protease	33	32	K,L2,L3,L4,L5,L6,L7,L8,phiNIT1,SP10
273	Tail Lysin	32	28	L,BanS-Tsamsa
277	Structural Protein	30	29	L,SP10
267	Baseplate	28	27	L
**Cell lysis proteins**				
282	Murein Transglycosylase	34	33	K1,L,0305φ8-36,SP10,BanS-Tsamsa
226	Holin	28	27	L
198	Holin	28	27	L
**Gene regulation/host functions**				
35	Bacterial SH3-like	29	28	D,F1,G,L5,L8,BanS-Tramsa,Doonfenshmirtz,ΦCM3
222	Metallophosphatase	28	27	L
260	cAMP Regulatory Protein	28	27	L
155	Beta Lactamase	26	25	L4,L5,L6,L7,L8,BanS-Tsamsa
787	Methyltransferase	20	19	L4,L5,L8
676	Sigma 70 Factor	20	19	L4,L5,L8

Abbreviations include deoxynucleotide monophosphate kinase (dNTP MonoP Kinase), ribonucleotide diphosphate reducatase (Ribonucleotide diP Reduct.), deoxyuridine nucleotidylhydrolase (dU Nucleotidylhydrolase), Methionine A-methyltransferase (Met A-methyltransferase), and major capsid protein (MCP). Gene products are given and are organized by basic function (DNA Replication/Metabolism, Virion Structure and Assembly, Cell Lysis Proteins, or Gene Regulation/Host Functions).

*Pham #’s are specific to this analysis and are larger than the total number of phams due to assignment by Phamerator [62].

**Table 5:** **Common** ***Bacillus*** **phage proteins of uncharacterized function with over 20 members**

Pham #*	# Members	# Phages	Phages (cluster or phage name)
**Uncharacterized proteins**			
154	40	23	L5,L6,L7,M8,Bc431v3
248	34	33	K,L,M,SP10
289	34	33	L3,L5,L7,M8,B4,Troll,Spock,Adelynn,BigBertha,Riley
4507	30	28	K,L1,L2,L4,M7
265	29	28	K1,L1,L2,L4,L5,L6,L7,M
288	29	28	I1,L,M
268	28	27	L,M
269	28	27	L,M
272	28	27	L,M
88	28	27	L,M
92	28	27	L1,L2,L3,L4,L5,L6,M
194	28	27	L,M
195	28	27	L,M
208	28	27	L,M
235	28	27	L,M
239	28	27	L,M
286	28	27	L,M
5235	27	27	L,M
302	27	27	L,M
5250	27	27	L,M
86	27	26	L,M
200	27	26	L,W.Ph.,Hakuna,Nagalana,Megatron,BPS10C
225	27	26	L1,L2,L3,L4,M7
228	27	26	L,M
255	27	26	L1,L2,L4,L5,L6,L7,M
287	27	26	L,W.Ph,Hakuna,Nagalana,BPS13,Megatron
296	27	26	L,W.Ph,Hakuna,Nagalana,BPS13,Megatron
5247	26	26	L4,L5,L6,L7,W.Ph.,Hakuna,Nagalana,Megatron,BPS10C
5240	26	26	L1,L4,L5,L6,L7,M
5190	26	27	L1,L2,L5,L6,L7,M
244	24	24	L5,L6,L7,M
251	24	23	L5,L6,L7,M
4539	24	22	J2,L5,L6,L7,M,PBC1
4492	24	24	L4,L5,L6,L7,M
4495	24	23	L4,L5,L6,L7,M8,Bc431
4496	23	22	L5,L6,M8,Bc431v
280	23	21	L5,L6,L7,M8
33	22	21	D,F,G,J2,K2,SP10,phBC6A51,BceA1
781	22	21	L5,L6,M
245	22	21	K2,Adelynn,BigBertha,Spock,Riley,BCP1,Hakuna,Nagalana, Megatron, Doofenshmirtz,
		-	Evoli, HoodyT, CAM003 ,IEBH ,JPB9
87	22	21	L1,L2,L3,L4,M7,Troll,Spock,Adelynn,BigBertha,Riley,L7,BPS10C,BPS13,
		-	Nagalana,Megatron,Bastille,Evoli,HoodyT,CAM003,Doofenshmirtz
176	20	20	L,M7,Bastille,CAM003,HoodyT,JPB9,Evoli
180	20	20	L,M7,Bastille,CAM003,HoodyT,JPB9,Evoli
618	20	19	L5,L6,M8
4500	20	22	L5,L6,M8
174	20	20	L5,L6,M8
4538	20	19	L5,L6,M8
303	20	20	L5,L6,M8
224	20	-	20 L5,L6,M8

*Pham #’s are specific to this analysis and are larger than the total number of phams due to assignment by Phamerator [62].

#### DNA replication/metabolism

The most highly conserved *Bacillus* gene product is ribonucleotide reductase (RNR), with homologs found in 41 of the 93 phages and six phages have multiple homologs. RNR forms deoxyribonucleotides from ribonucleotides for DNA biosynthesis and is commonly found in lytic phages [77]. Other well-conserved proteins for nucleotide metabolism include a dihydrofolate reductase (conserved in 31 phages), thymidylate synthase (conserved in 28 phages), dNTP monophosphate kinase (conserved in 29 phages), ribonucleotide deoxyphosphate reductase (conserved in 27 phages) and a glutaredoxin (conserved in 24 phages). Many putative proteins involved in DNA replication and recombination were also identified including a DNA helicase (conserved in 33 phages), replicative helicase (conserved in 27 phages), DNA exonuclease and endonuclease (conserved in 33 and 32 phages, respectively), DNA polymerase (conserved in 27 phages), RecA homolog (conserved in 27 phages), and a DNA primase (conserved in 27 phages). These results underscore the vital nature of efficient nucleotide metabolism in the propagation of lytic phages .

#### Virion structure and assembly proteins

The structural and assembly proteins of the virion are also highly conserved gene products within the *Bacillus* phages, with phams consisting of a MCP, large terminase, portal protein, capsid structural protein, baseplate, tail sheath, and a tail lysin all having homologs in 33 of the 93 phages (35%). In addition, a procapsid protease, tail fiber, tail assembly chaperone, virion structural protein and a baseplate have homologs in at least 27 of the 93 phages. These structural proteins are conserved among phages that are known myoviruses and siphoviruses, although the podoviruses and tectiviruses should also contain an MCP, portal protein and terminase. We were able to identify a large terminase for all of the *Bacillus* phages, meaning that these gene products had homologs that were somewhat characterized, but not homologous to the prevalent Pham. In contrast, we were unable to identify an MCP for 19% of the *Bacillus* phages, suggesting that homologs have not been described and emphasizing the need for further characterization of *Bacillus* phages. In support of this finding, recent studies have shown that MCP’s bearing no amino acid sequence similarity can harbor similar folds [21, 22, 78–80] hampering identification by sequence alone.

#### Cell lysis

Cell lysis proteins are vital to the phage lifecycle, allowing them to exit the cell and infect other hosts. Three cell lysis proteins were well-conserved including a murein-transglycosylase (conserved in 33 phages) and two holins (each conserved in 27 phages).

#### Host functions/pathogenesis

Several gene products that are likely to regulate host functions were also highly conserved in *Bacillus* phages. A protein containing a bacterial SH3-like domain was identified in 28 of the 93 phages, including phages from cluster D, F, G, and L. The function of this protein is unknown but the SH3 domain is thought to mediate the assembly of large multiprotein complexes [23]. In addition, the cAMP regulatory protein (CRP) is found in 27 phages and a sigma-70 factor in 19 phages, which may both be used to control the expression of host carbon metabolism genes which can contribute to bacterial virulence [81]. An FtsK/SpoIIIE-like cell division protein (gp22 in phage Cherry) was conserved in 24 of the phages (pham370). This protein may control host transition into the sporulation state, contributing to the environmental fitness of *B. anthracis* [29].

There are several other proteins that are less conserved that may contribute to host pathogenesis. Three *Bacillus* phages (JL, Shanette and SP10) harbor a dUTPase, which are common in many bacteriophages and have been shown to function as G protein-like regulators required for the transfer of staphylococcal virulence factors [82, 83]. Five *Bacillus* phages (SPO1, CampHawk, Pegasus, JL, and Shanette), encode a Pho-H like protein that aids in bacterial survival under phosphate starvation [84, 85]. Genes belonging to the phosphate regulon are reportedly very common in marine phages (40%) while they are less common in non-marine phages (4%) [86], in good agreement with our identification of PhoH in 5.4% of the *Bacillus* phages.

Subcluster F1 phages encode resistance to the soil antibiotic fosfomycin, which may account for the resistance reported for *B. anthracis* strains [29]. In addition, JL and Shanette both encode the tellurium resistance proteins TerE and TerC. Tellurium oxyanion (TeO_3_^2−^) has been used in the treatment of mycobacterial infections and resistance is a feature of many pathogenic bacteria. In fact, resistance is commonly used for the identification and isolation of Shiga toxin-producing *E. coli* [87].

### The comparison of subcluster and bacterial host reveals evolutionary boundaries

The *Bacillus* hosts in this study can be assembled into two separate groups by relatedness, and this evolutionary boundary may define phage boundaries and predict barriers for pathogenic gene transfer. *B. subtilis*, *B. megaterium* and *B. pumulis* are more closely related to each other than they are to the Bacillus cereus group of bacteria, comprised of *B. cereus*, *B. anthracis*, *B. thuringiensis*, *B. weihenstephanensis, B. mycoides* and *B. pseudomycoides* [88, 89]. To determine if there are such boundaries between phages and their hosts, the host from which each phage was isolated was compared within each cluster and subcluster.

The cluster to bacterial host relationship was somewhat ambiguous, with 67% of clusters populated by phages from only closely related *Bacillus* species (clusters A, B, C, D, E, F, G, and I) and others (clusters H, J, K and L) harboring phages from more distantly related *Bacillus* species (see Table 2). However, within these latter clusters there is a clear division at the subcluster level in that *B. subtilis*, *B. pumulis,* and *B. megaterium* phages *always* fall into a separate subcluster than phages that infect *B. cereus*, *B. thuringiensis*, *B. anthracis,* and *B. weihenstephanensis*. More phages are clearly needed to understand the host diversity within clusters, however, because only four clusters contain phages from diverse hosts (phages from both a *B. subtilis*, *B. pumulis*, *B. megaterium* host and from a Bacillus cereus group host). In addition, this analysis was performed using only the host from which the phage was isolated since the host range of most of these phages is unknown. Host range studies will provide greater insight. For example, a recent finding that phage BPC78 infects both *B. cereus and B. subtilis* suggests that some phages are able to overcome this apparent host boundary [44].

The subcluster to host analysis also suggests a closer relationship between the *B. thuringiensis* and *B. cereus* species when compared to *B. anthracis*, since there is a subcluster division between *B. anthracis* phages and those that infect *B. thuringiensis* or *B. cereus* (see Figure 2, clusters A and F). This apparent evolutionary separation is surprising given the recent report of five phages that infect *B. anthracis* and *B. thuringiensis* as well as the *B. cereus* host on which they were isolated (BanS-Tsama [59], Bc431v3 [90], and JL, Shanette, and Basilisk [21]).

## Conclusions

Phages are intimately linked to the ecology and evolution of their hosts, making phage characterization vital to understanding the diversity and evolution of the *Bacillus* genus. Herein we described the comparison of 93 fully sequenced *Bacillus* phages and their grouping into 12 clusters, 14 singletons and 28 subclusters (see Tables 1 and 2). In addition, two groups of more distantly-related phages were identified and termed “superclusters”, namely the SPO1-like and d’Herelle-like phages. This analysis of *Bacillus* phages may aid in understanding newly isolated phages as well as the enormous complexity of tailed phages. It may also serve as a reference for comparisons to phages that infect other genera. The only other such analyses are of 491 phages that infect *Mycobacterium* and of 337 phages that infect the *Enterobacteriaceae* family. Hatfull et al. grouped the Mycobacteriophages into ~17 “clusters” of related phages (A-Q) and 14 singleton clusters [13], while Grose and Casjens grouped the *Enterobacteriaceae* phages into 38 clusters of related phages and 18 singleton clusters [14]. In contrast to both of these phage groups, the *Bacillus* singletons outnumber the *Bacillus* clusters, presumably due to the decreased number of total phages isolated (93 phages as compared to 491 or 337). It should also be noted that additional *Bacillus* phage isolation will most likely require future revision of these cluster assignments as phages may be isolated that unite clusters.

Our analysis revealed several clusters of highly related phages (clusters C, D, E, H and I), and other clusters that contained very diverse phages (A, B, F, G, J, K and L) (see Figures 2 and 3). Due to the low number of phages isolated and the apparent expected diversity, it is currently unknown if these differences reflect differences in phage lifestyles, or if they occur due to sampling biases. Our analysis also revealed the need for using several analytical techniques to group phages, since one technique may suggest apparent relatedness that is weak by other techniques. For example, the B and C clusters share ~29% proteome conservation as analyzed by CoreGenes and faint lines of similarity can be seen in genomic dot plots. However, analysis of the overall genome synteny suggests they are more diverse in lifestyle than phages that typically form clusters/superclusters (see Figure 6).

In addition to whole genome analysis, analysis of *Bacillus* phage gene products further underscores the enormity of *Bacillus* phage diversity, with 81% of protein phams (3,971) consisting of uncharacterized proteins. In addition, ~19% of MCPs were unannotated and unidentifiable, highlighting the uncharacterized nature of these phages. Since several phams of known function were identified that may contribute to host pathogenicity, understanding these uncharacterized phams is critical to understanding the evolution of pathogenic *Bacillus* strains.

The analysis of *Bacillus* phage evolutionary boundaries suggests that close phage relationships (defined by subclusters) are restricted by the relatedness of the host, with the phages that infect the Bacillus cereus group of phages more similar than those that infect *B. subtilis*, *B. megaterium* and *P. pumulis*. This analysis of host vs. cluster is not only beneficial to understanding the evolution of *Bacillus* species but may indicate phage clusters more suitable for targeted phage therapy of pathogenic *B. cereus* and *B. anthracis* strains.

## Methods

### Computational analysis and genomic comparison

*Bacillus* phage sequences were retrieved from GenBank and the *Bacillus* Phage Database at PhagesDB.org as well as by contact with the authors of this website. To ensure retrieval of all *Bacillus* phages from GenBank, the major capsid protein (MCP) from at least one phage in each cluster was used to retrieve all phages with similar MCP sequence via TBLASTN [91]. Genomic maps of each phage were prepared using Phamerator [62], an open-source program designed to compare phage genomes. Phamerator was also used to calculate the percent G/C, number of ORFs and protein families or phams. The percentage of the proteome conserved was identified using the program CoreGenes 3.0 at the default BLASTP threshold of 75 [63, 64], while average nucleotide identity (ANI) was calculated by Kalign [92]. Dot plots were generated using Gepard [61]. For ease in dot plot analysis, long direct terminal repeats were removed from some phages, other phage genomes were reverse complemented, and new bp one calls were made to re-orient according to the majority of phages within a cluster. In addition, a portion of the PZA nucleotide sequence was reverse complemented to allow alignment with other phages of the cluster. Whole genome amino acid sequences were retrieved from Phamerator [62].

The terminase phylogenetic tree was constructed using a MUSCLE [69] alignment and the neighbor-joining method in Mega5 [70]. Bootstrapping was set to 2000 and the unrooted tree was collapsed at a less than 50% bootstrap value. Sequences for comparison were chosen by annotated large terminase gene products or a BlastP alignment to the closest relative when unannotated.

## Abbreviations

B: *Bacillus sp*
*Tectiviridae* (T): Viral family abbreviations
P: *Podoviridae*
S: *Siphoviridae*
M: *Myoviridae*
MCP: Phage protein abbreviations: Major Capsid Protein
TMP: Tape Measure Protein
ANI: Other: Average Nucleotide Identity
bp: Base pair
kbp: Kilobase pair
ORF: Open Reading Frame
pham: Phage protein family identified by Phamerator
UK: Unknown.
