# Erratum to: genomic comparison of 93 *Bacillus* phages reveals 12 clusters, 14 singletons and remarkable diversity

**DOI:** 10.1186/1471-2164-15-1184

**Published:** 2014-12-29

**Authors:** Julianne H Grose, Garrett L Jensen, Sandra H Burnett, Donald P Breakwell

**Affiliations:** Microbiology and Molecular Biology Department, Brigham Young University, Provo, UT USA

**Keywords:** Bacteriophage, Phage, Cluster, *Bacillus*

## Abstract

**Background:**

The *Bacillus* genus of Firmicutes bacteria is ubiquitous in nature and includes one of the best characterized model organisms, *B. subtilis,* as well as medically significant human pathogens, the most notorious being *B. anthracis* and *B. cereus.* As the most abundant living entities on the planet, bacteriophages are known to heavily influence the ecology and evolution of their hosts, including providing virulence factors. Thus, the identification and analysis of *Bacillus* phages is critical to understanding the evolution of *Bacillus* species, including pathogenic strains.

**Results:**

Whole genome nucleotide and proteome comparison of the 83 extant, fully sequenced *Bacillus* phages revealed 10 distinct clusters, 24 subclusters and 15 singleton phages. Host analysis of these clusters supports host boundaries at the subcluster level and suggests phages as vectors for genetic transfer within the *Bacillus cereus* group, with *B. anthracis* as a distant member. Analysis of the proteins conserved among these phages reveals enormous diversity and the uncharacterized nature of these phages, with a total of 4,442 protein families (phams) of which only 894 (20%) had a predicted function. In addition, 2,583 (58%) of phams were orphams (phams containing a single member). The most populated phams were those encoding proteins involved in DNA metabolism, virion structure and assembly, cell lysis, or host function. These included several genes that may contribute to the pathogenicity of *Bacillus* strains.

**Conclusions:**

This analysis provides a basis for understanding and characterizing *Bacillus* and other related phages as well as their contributions to the evolution and pathogenicity of *Bacillus cereus* group bacteria. The presence of sparsely populated clusters, the high ratio of singletons to clusters, and the large number of uncharacterized, conserved proteins confirms the need for more *Bacillus* phage isolation in order to understand the full extent of their diversity as well as their impact on host evolution.

## Correction

The version of this article published in BMC genomics 2014 15(1):855, contains unpublished genomes downloaded from the public website phagesdb.org. We apologize for not having contacted the authors of these genomes in advance. In this correction, we removed all unpublished genomes as of the original publication date at authors request (Adelynn, Doofinshmertz, Gir1, JPB9, Nigalana Polaris, Pleiades, Pappano, Pegasus, Stitch). Removing these data did not alter the principle results and conclusions of our original work, including conservation of 100% the phage relationships (grouping into clusters and subclusters). It only altered their numbers, with 83 total phages, 10 clusters and 15 singletons. Hence the figures, tables and text are very similar, with minor changes in numbering and wording. In addition, we have repaired and updated some of the references for Table 1. We apologize for any confusion or inconvenience this may have caused.

## Background

Bacteriophages are the most abundant biological entities on the planet, with at least 10^31^ bacteriophages in Earth’s biosphere [1–5]. Their ability to infect and kill their bacterial hosts makes them key factors in both the evolution of bacteria and the maintenance of ecological balance (for recent reviews see [6–12]). In addition, they are able to infect and transfer genetic information to their hosts, in many cases being key factors in the transfer of pathogenic traits such as in pathogenic *Escherichia coli*, *Salmonella sp., Corynebacterium diphtheriae* and *Vibrio cholerae*. Despite their clear importance to global environmental and health concerns, little is known about the complexity and diversity of these living entities, but what is known from metagenomics and phage genome sequencing suggests it is vast.

The most studied bacteriophages are those that infect the Gram-positive bacterium Mycobacterium smegmatis mc^2^155, with over 4,800 phages isolated and 690 fully sequenced genomes (http://www.phagesdb.org). These phages have been isolated by students from throughout the world as part of the Howard Hughes Medical Institute Science Education Alliance Phage Hunters Advancing Genomics and Evolutionary Science (HHMI SEA-PHAGES) for determining the diversity of phages that can infect a single host. A recent analysis of 491 of these indicates they belong to approximately 17 “clusters” of related phages (A-Q) and 13 singleton clusters [13]. Of interest, identical mycobacteriophages have only been isolated independently twice (Graham Hatfull, personal communication). Beyond these *Mycobacterium* phages, the bacterial family with the most phages isolated is the Gram-negative *Enterobacteriaceae* family (337 fully sequenced genomes available in GenBank). This group of phages has been isolated and sequenced independently from investigators throughout the world and contains many of the well-characterized, historical phages such as Lambda, Mu, T4 and T7. They have recently been grouped into 38 clusters of related phages and 18 singleton clusters [14].

A third group of well-studied phages, the *Bacillus* phages, have also been isolated by diverse investigators from throughout the world and infect many strains of the genus *Bacillus*. The *Bacillus* genus is ubiquitous in nature and includes one of the best characterized model organisms, *B. subtilis,* as well as medically significant human pathogens, the most notorious being *B. anthracis* (the causative agent of anthrax) and *B. cereus* (which causes food poisoning). Phages have been isolated that infect *B. anthracis*, *B. cereus*, *B. megaterium, B. mycoides, B. pseudomycoides, B. subtilis, B. thuringiensis*, and *B. weihenstephanensis*, allowing a unique opportunity to investigate the diversity of phages that infect different hosts within a bacterial genus. This study focuses on the genomic comparison of 83 fully sequenced phages that infect the *Bacillus* genus and discusses their place in the diversity and evolution of these important bacteria. In addition, we identify several genes that may contribute to the pathogenicity of *Bacillus* species. This analysis presents a framework for understanding phages that infect *Bacillus* and for comparing *Bacillus* phage diversity with the diversity of phages that infect other genera. In addition, it increases our understanding of the evolution and diversity of phages and their hosts, including the evolution of pathogenic strains.

## Results and discussion

### Whole genome nucleotide and amino acid comparison of the Bacillus family of phages reveals 10 diverse clusters of related phages and 15 singleton clusters

In order to determine the relationship of the 83 extant, fully-sequenced *Bacillus* phages, we analyzed the published phage genomes by methods similar to those of Hatfull et al. [15, 16], including whole genome dot plot analysis, pairwise average nucleotide identities (ANI) and genomic maps. The accession numbers and basic properties (host, genome size, GC content, number of ORFs, number of tRNAs and morphotype) of the 83 fully sequenced *Bacillus* phages are provided in Table 1 along with the appropriate reference.

**Table 1:** **Characteristics of reported** ***Bacillus*** **phages with complete genome sequences**

Sub.	Phage Name	Host	Size (bp)	GC%	ORFS	tRNA	Accession #	Family	Ref.
A1	***Wip1***	A	14319	36.84	27	0	NC_022094	*T*	[60]
A1	AP50	A	14398	38.65	31	0	NC_011523	*T*	[61]
A2	GIL16c	T	14844	39.72	32	0	NC_006945	*T*	[62]
A2	Bam35c	T	14935	40.08	31	0	AY257*527*	*T*	[63]
A2	pGIL01	T	14931	39.73	30	0	AJ536073	*T*	[64]
B1	***Phi 29***	S	19282	39.99	27	0	EU771092	*P*	
B1	PZA	S	19366	39.66	27	0	M11813	*P*	[65]
B2	B103	S	18630	37.66	17	0	NC_004165	*P*	[36]
B2	Nf	S	18753	37.32	27	0	EU622808	*P*	[66]
B3	GA-1	B	21129	34.66	35	1	X96987	*P*	[67]
C1	***Pony***	M	39844	40.70	48	0	*NC_022770*	*P*	[68]
C1	Poppyseed	M	39874	40.71	50	0	KF669657		
C1	Page	M	39874	40.71	50	0	*NC_022764*	*P*	[69]
D1	***TP21-L***	T	37456	37.80	56	0	NC_011645	*S*	[70]
D1	BMBtp2	T	36932	37.79	53	0	NC_019912	*S*	[71]
D1	ProCM3	T	43278	37.36	66	0	KF296717	*S*	[72]
E1	***γ d’Herelle***	A	37373	35.13	53	0	DQ289556	*S*	[43]
E1	γ isolate 51	A	37253	35.22	53	0	DQ222853	*S*	[73]
E1	Wβ	A	40867	35.26	53	0	DQ289555	*S*	[43]
E1	Gamma	A	37253	35.22	53	0	NC_007458	*S*	[73]
E1	Cherry	A	36615	35.27	51	0	DQ222851	*S*	[73]
E1	γ isolate 53	A	38067	35.10	50	0	DQ222855	*S*	[74]
E1	Fah	A	37974	34.95	50	0	NC_007814	*S*	[74]
E2	phiCM3	T	38772	35.48	56	0	NC_023599	*S*	[71]
E2	phIS3501	T	44401	34.86	53	1	JQ062992	*S*	[75]
E2	BtCS33	T	41992	35.22	57	0	NC_018085	*S*	[72]
E3	BceA1	C	42932	35.66	63	0	HE614282	*S*	[76]
F1	***IEBH***	C	53104	36.42	86	0	EU874396	*S*	[76]
F1	250	C	56505	36.44	54	0	GU229986	*S*	[77]
G1	***Andromeda***	P	49259	41.91	79	0	NC_020478	*S*	[18]
G1	Gemini	P	49362	41.9	79	0	KC330681	*S*	[18]
G1	Glittering	P	49246	42.05	78	0	NC_022766	*S*	[78]
G1	Curly	P	49425	41.82	77	0	NC_020479	*S*	[18]
G1	Eoghan	P	49458	42.21	76	0	NC_020477	*S*	[18]
G1	Taylor	P	49492	42.29	75	0	KC330682	*S*	[18]
G1	Riggi	P	49836	41.46	79	0	NC_022765	*S*	[79]
G1	Blastoid	P	50354	42.23	79	0	NC_022773	*S*	[80]
G1	Finn	P	50161	41.69	77	0	NC_020480	*S*	[18]
H1	Staley	M	81656	35.35	113	0	*NC_022767*	*S*	[81]
H1	Slash	M	80382	35.23	112	0	*KF669661*	*S*	[82]
H2	Basilisk	C	81790	33.9	141	2	KC595511	*S*	[83]
I1	***SPO1***	S	132562	39.97	204	5	NC_011421	*M*	[84]
I1	CampHawk	S	146193	40.2	229	2	NC_022761	*M*	[85]
I2	Shanette	C	138877	40.8	223	3	KC595513	*M*	[83]
I2	JL	C	137918	40.8	222	4	KC595512	*M*	[83]
J1	***PhiNIT1***	S	155631	42.12	215	4	NC_021856		[86]
J1	Grass	S	156648	42.25	242	3	NC_022771	*M*	[87]
J2	SI0phi**	S	146698	39.02	206	0	KC699836	*M*	
J3	phiAGATE	P	149844	49.97	204	4	NC_020081	*M*	[88]
J4	Bastille	T	153962	38.14	273	7	JF966203	*M*	[29]
J4	Evoli	T	159656	38.06	294	8	KJ489398	*M*	
J4	Hoody T	T	159837	38.01	270	8	KJ489400	*M*	
J4	CAM003	T	160541	38.03	287	8	KJ489397	*M*	
J5	B4	C	162596	37.71	277	0	JN790865	*M*	[89]
J5	Troll	T	163019	37.83	289	0	NC_022088	*M*	
J5	Spock	T	164297	37.62	283	0	NC_022763	*M*	
J5	BigBertha	T	165238	37.77	287	0	NC_022769	*M*	[90]
J5	Riley	T	162816	37.78	290	0	NC_024788	*M*	
J5	B5S	C	162598	37.71	272	0	JN797796	*M*	[17]
J6	BCP78	C	156176	39.86	227	18	JN797797	*M*	[52]
J6	BCU4	C	154371	39.86	223	19	JN797798	*M*	[17]
J7	BCP1	C	152778	39.76	227	17*	KJ451625	*M*	
J7	Bc431v3	C	158621	39.98	239	21	JX094431	*M*	[54]
J8	Hakuna	T	158100	38.70	294	0	KJ489399H	*M*	
J8	Megatron	T	158750	38.80	290	0	KJ4894011H	*M*	
J8	BPS10C	C	159590	38.74	271	0	NC_023501	*M*	[91]
J8	BPS13	C	158305	38.75	268	0	JN654439	*M*	[91]
J8	W.Ph.	C	156897	36.45	274	0	HM144387	*M*	[29]
Single	MG-B1	W	27190	30.75	42	0	NC_021336	*P*	[26]
Single	BV1	B	35055	44.85	54	0	DQ840344		
Single	phBC6A52	C	38472	34.72	49	0	NC_004821		[72]
Single	phi105	S	39325	42.69	51	0	NC_004167	*S*	[92]
Single	BCJA1C	Bc	41092	41.74	59	0	NC_006557	*S*	[84]
Single	PBC1	C	41164	41.68	50	0	JQ619704	*S*	[93]
Single	SPP1	S	44010	43.72	81	0	NC_004166	*S*	[94]
Single	PM1	S	50861	41.29	86	0	NC_020883	*S*	[95]
Single	phBC6A51	T	61395	37.69	75	0	NC_004820		[72]
Single	BCD7	C	93839	38.04	140	0	JN712910	*M*	
Single	SPBc2	S	134416	34.64	185	0	NC_001884	*S*	[96]
Single	SP10	S	143986	40.49	236	0	NC_019487	*M*	[33]
Single	BanS-Tsamsa	A	168876	34.32	272	19	NC_023007	*S*	[53]
Single	0305phi8-36	T	218948	41.8	247	2	NC_009760	*M*	[97]
Single	G	M	497513	29.93	675	18	JN638751	*M*	

The subcluster and cluster designation is given followed by the phage name. The founding phage for each cluster is in bold-italics. Hosts are the bacterial hosts on which the phages were isolated (not the host range) and are abbreviated as *Bacillus anthracis* (A), *Bacillus cereus* (C), *Bacillus sp.* (B), *Bacillus clarkii* (Bc), *Bacillus megaterium* (M), *Bacillus pumilus* (P), *Bacillus subtilis* (S), *Bacillus thuringiensis* (T), and *Bacillus weihenstephanensis* (W). ORFs are the number of Open Reading Frames predicted to be encoded by the genome as provided in the reported annotation. Family is *Myoviridae* (M), *Siphoviridae* (S) or *Podoviridae* (P). A reference (Ref) for the GenBank sequence is provided when available.

*tRNA predicted in this study using Aragorn and DNAMaster.

**Phage SI0phi is reported as an incomplete genome but is included in this analysis because it was complete enough to clearly assign it to a cluster.

Dot plot analysis of the *Bacillus* phages revealed 10 clusters of phages with similarity over at least 50% of their genomes (clusters A-J, also referred to by “founding phage” for clarity) and 15 phages that are singletons, having little to no nucleotide similarity to any other *Bacillus* phages. Genomic dot plot analysis consists of placing the nucleotide sequences across both the X- and Y-axis. A dot is placed where the sequences are identical, resulting in a diagonal line down the center of the plot when a sequence is compared to itself. The phages were aligned on two separate plots due to the wide range in genome size and the fact that no additional nucleotide similarity was seen in a combined plot. Figure 1A contains phage genomes of less than 100 kb while 1B contains the larger phage genomes. As stated above, assignment of a phage to a cluster was based on nucleotide similarity over at least 50% of the genome when compared to at least one other phage in the cluster. A phage could be placed into the same cluster by weak similarity over most of the genome, by strong similarity over about half of the genome, or by a combination of relatedness. The ANI values were also calculated within each cluster and found to be at least 55% between a phage and another phage within a cluster. From the total of 25 clusters over half (15) are singleton clusters containing a single phage member, suggesting that the isolation of unique *Bacillus* phages is far from complete. Our analysis and grouping of phages into clusters agrees completely with a previous grouping of *B. cereus* group phages by Lee et al. in which our PhiNIT1-like J cluster phages would belong to Group I, our gamma d’Herelle-like E cluster phages to Group II, and our Wip1-like A cluster phages to Group III [17]. In addition it agrees with the recent grouping of *B. pumilus* phages into BpA, where BpA corresponds to our A cluster [18].

**Figure 1:**
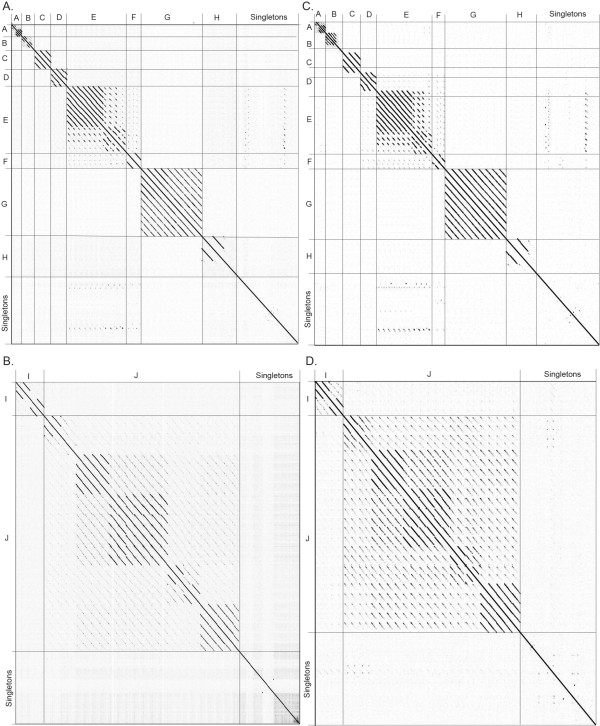
**Nucleotide and amino acid dot plot analysis of 83 fully sequenced** ***Bacillus*** **phages reveals 10 clusters (A-J) and 15 singletons.** Nucleotide dot plot of *Bacillus* genomes of less than **(A)** or greater than **(D)** 100 kb organized by similarity reveals 10 clusters of related phages. Amino acid dot plot of *Bacillus* genomes of less than **(C)** or greater than **(D)** 100 kb organized by similarity reveals 10 clusters of related phages. Thick lines indicate cluster assignments, which are provided on the Y-axis **(A-J)**. Dot plots were produced using Gepard [57] and whole genome amino acid sequences were retrieved from Phamerator [34].

In addition to showing strong evolutionary relationships, whole genome nucleotide dot plots also reveal smaller regions of homology (<50% span length) between phages of different clusters that are likely areas of recombination. The largest such region is a ~10,000 bp region of similarity between phBC6A51 (bp 44289–50616 and 58088–61389) and gamma d’Herelle-like E cluster phages that includes a tail component protein, minor structure protein and holin as well as a site-specific recombinase, a Ftsk/SpoIIIE family protein and five conserved phage proteins.

In addition to whole genome nucleotide analysis, whole proteome dot plot analysis was performed (Figures 1C and D). Because nucleotide sequences diverge more rapidly, the amino acid dot plots were expected to reveal more distant evolutionary relationships. The analysis confirmed the basic cluster assignments seen with whole genome nucleotide analysis and revealed distant relationships between the TP21-like D, gamma d’Herelle-like E, and IEBH-like F cluster phages discussed in more detail below. Note that there should be some limited similarity between all of the *Bacillus* tailed phages in that they should all encode a major capsid protein (MCP), portal protein and terminase. However, these proteins can diverge to a point that no sequence similarity is apparent.

Another common way to group phages is by the percent of the proteome that is conserved between phages. CoreGenes 3.0 was used to confirm clusters by ensuring that phages within a cluster share ~40% of their proteome, a cutoff commonly used for determining phage relationships [19, 20]. The cluster with the lowest conservation of the proteome (that is, the lowest conservation between a phage and its closest relative) is the Staley-like H cluster, with the highly related phages Staley and Slash sharing only 43.4% of their proteome with Basilisk. All other clusters yielded proteome comparison scores well above the 40% CoreGenes threshold, thus confirming that the phages belong in the proposed clusters.

The division of phages into the proposed clusters is also supported by the low standard deviation in the average basic phage properties including genome size, GC content, number of ORFs and morphotype (Table 2). For example, the cluster A consists completely of tectiviruses of an average genome size of 14685 ± 302 bp, clusters B and C of podoviruses with short tails (average genome size is 19432 ± 1001 and 39864 ± 17 bp, respectively), clusters D, E, F, G and H of long noncontractile siphoviruses (average genome size ranging from 39222 ± 3522 to 81276 ± 777 bp), and the large contractile myovirus clusters I and J (average genome size is 138886 ± 5607 and 158129 ± 4580 bp, respectively). The average number of tRNA’s for each cluster is also reported but is highly variable within a cluster, with standard deviations often approaching the number of tRNAs. This variation may reflect the phages’ adaptation to different hosts because tRNAs are thought to provide efficient protein production in hosts with alternate codon preferences [21]. Further host range studies are needed to test these hypotheses.

**Table 2:** **Summary of** ***Bacillus*** **cluster phage characteristics**

Cluster	Sub.	Phages	Hosts	Genome size	% GC	# ORFS (tRNA)	Type
A	2	5	A, T	14685 ± 302	39.0 ± 1,3	30.2 ± 1.9(0)	T
B	3	5	B, S	19432 ± 1001	37.9 ± 2.1	26.6 ± 6.3(0.3 ± 0.6)	P
C	1	3	M	39864 ± 17	40.7 ± 0.0	49.3 ± 1.2(0)	P
D	1	3	C, T	39222 ± 3522	37.7 ± 0.3	48.7 ± 10.2(0)	S
E	3	11	A, C, T	39409 ± 2677	35.2 ± 0.2	53.8 ± 3.7(0.09 ± 0.3)	S
F	1	2	C	54805 ± 2405	36.4 ± 0.0	70.0 ± 22.6(0)	S
G	1	9	M, P	49621 ± 402	42.0 ± 0.3	77.7 ± 1.5(0)	S
H	2	3	C, M	81276 ± 777	34.8 ± 0.8	122 ± 16.8(0.7 ± 1.2)	S
I	2	4	C, S	138886 ± 5607	40.4 ± 0.4	220 ± 10.8(3.5 ± 1.3)	M
J	8	23	C, P, S, T	158129 ± 4580	39.3 ± 2.7	261.0 ± 30.4(4.5 ± 6.6)	M

Characteristics given are cluster assignment, number of subclusters (Sub.), number of phages in the cluster, host species from which the phages were isolated, the average genome size, average percent GC content, average number of ORFS with average number tRNA in parenthesis, and the morphotype. Averages are given with the standard deviation. Species abbreviations are *Bacillus anthracis* (A), *Bacillus cereus* (C), *Bacillus sp.* (B), *Bacillus megaterium* (M), *Bacillus pumilus* (P), *Bacillus subtilis* (S), *Bacillus thuringiensis* (T), and *Bacillus westenstephanensis* MG1, (W). Family/ Morphotype abbreviations are *Tectiviridae* (T), *Podoviridae* (P), *Siphoviridae* (S), and *Myoviridae* (M).

### Division of clusters into subclusters reveals large variance between clusters

Each cluster was further analyzed by nucleotide dot plot to reveal groups of high similarity, or subclusters (Figures 2 and 3). These subclusters were chosen based on natural divisions in phage similarity seen in the dot plot, but could be more strictly defined by ANI values of at least 66% between two phages within the subcluster. The subcluster assignments indicate great diversity in the relatedness within each *Bacillus* phage cluster. It is unknown whether this diversity represents evolutionary forces that constrain certain types of phages or if it is an artifact of phage isolation. Further phage isolation is necessary for this distinction.

**Figure 2:**
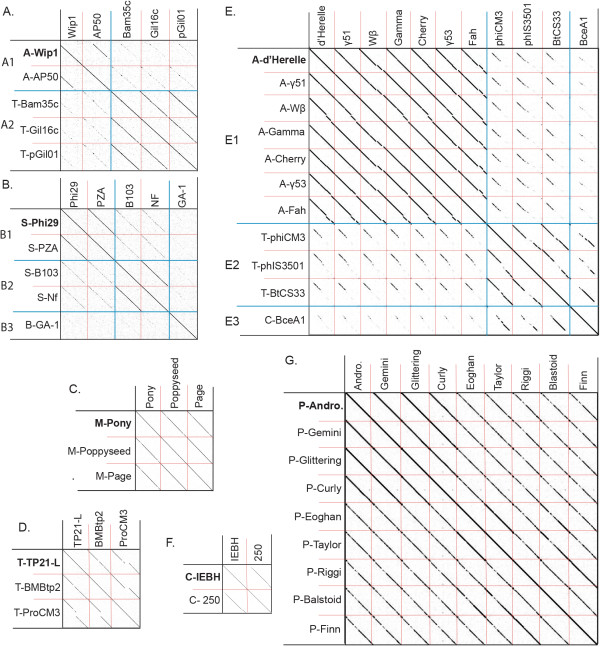
**Analysis of fully sequenced** ***Bacillus*** **phage genomes belonging to clusters A through G reveals 12 subclusters.** Subcluster divisions are provided by blue lines and are indicated on the Y-axis when there are more than one per cluster. Individual phages are separated by red lines. Phage names are provided on the X-axis and Y-axis with host abbreviation from which the phages were isolated indicated on the Y-axis. The founding phage for each cluster is bolded. Hosts abbreviations are *Bacillus anthracis* (A), *Bacillus cereus* (C), *Bacillus sp*. (B), *Bacillus megaterium* (M), *Bacillus pumilus* (P), *Bacillus subtilis* (S), *Bacillus thuringiensis* (T), and *Bacillus weihenstephanensis* MG1, (W). Dot plots were produced using Gepard [57]. Phage Andromeda is abbreviated (Andro.).

**Figure 3:**
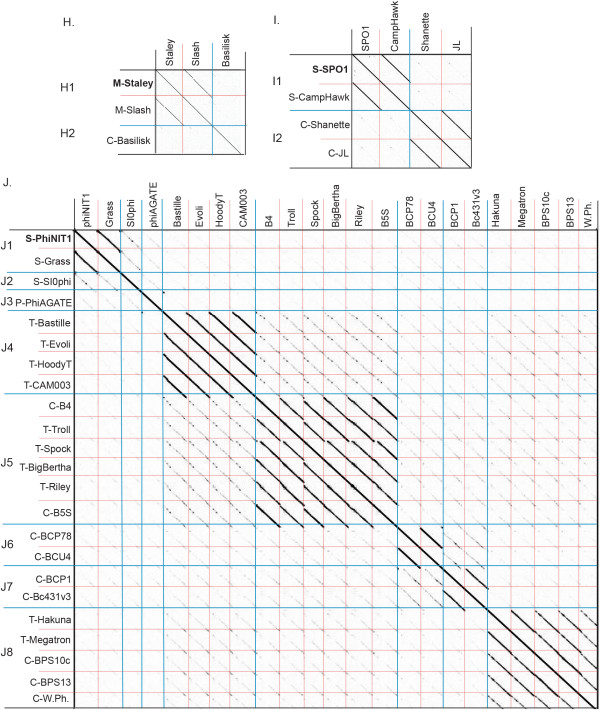
**Analysis of fully sequenced** ***Bacillus*** **phage genomes belonging to clusters H through J reveals 12 subclusters.** Subcluster divisions are provided by blue lines and are indicated on the Y-axis when there are more than one per cluster. Phages are separated by red lines. Phage names are provided on the X-axis and Y-axis with host abbreviation from which the phages were isolated provided first. The founding phage for each cluster is bolded. Hosts abbreviations are *Bacillus anthracis* (A), *Bacillus cereus* (C), *Bacillus sp.* (B), *Bacillus megaterium* (M), *Bacillus pumilus* (P), *Bacillus subtilis* (S), *Bacillus thuringiensis* (T), and *Bacillus weihenstephanensis* MG1, (W). Dot plots were produced using Gepard [57].

### Clusters containing highly related phages

Clusters C, D, and F and G are each comprised of a single subcluster containing highly related phages (sharing at least 74% ANI). Cluster G is the largest cluster containing only highly related phages, and harbors 9 myovirus phages [18], the clusters C and D each contain three phages of the podovirus and siphovirus families, respectively, while F has two siphoviruses. The majority of phages in each of these clusters are recently isolated phages that are not well characterized. In fact, the MCP was not annotated for any cluster C or D phage and we were unable to identify an MCP by TBLASTN searches, suggesting that the MCP of these phages are novel.

### Clusters containing more distantly related phages

Clusters A, B, E, H, I and J all contain multiple subclusters, with B, E, H and J being the most variable. Cluster B contains three subclusters having ANI values ranging from 48% to 76% between phages (but all phages have at least 54% with at least one other phage in a different subcluster). A CoreGenes 3.0 analysis confirms this relationship of cluster B phages, with B1 phages sharing 96% of their proteome within the subcluster but approximately 63% and 56% with the B2 and B3 cluster proteomes, respectively. This family of phages has been previously referred to as the Phi 29 family, and our subclustering pattern is consistent with the analysis and grouping done by Pecenkova and Paces [22]. Similarly, cluster E contains 11 phages divided into 3 subclusters where ANI varies from 42% to 99.99% between phages but all phages have at least 55% to one another. There is 86% proteome conservation within each subcluster, and between subclusters there is at least 41% proteome conservation. Cluster H harbors the very similar Staley and Slash (94% ANI) and the more distantly related phage Basilisk, which shares ~55% ANI and 43% of its proteome with Staley/Slash. Cluster I harbors SPO1 and close relatives CampHawk (subcluster I1) as well as the more distantly related phages Shanette and JL (subcluster I2), which share ~53% of their proteomes with the I1 phages.

Clusters F and J contain more closely related phages. Cluster F harbors siphoviruses IEBH and 250 which share 90% ANI and 55% of their proteomes. Cluster J is the largest cluster and contains 23 myoviruses. Of interest, the eight subclusters to which these large phages belong are highly variable in host, tRNA content and number of ORF’s (see Table 1), but they are all highly related having at least 81% ANI.

Overall, *Bacillus* phages remain highly uncharacterized but clusters B, E and I contain a some of well characterized *Bacillus* phages including the *B. subtilis* phage phi 29, the *B. anthracis* typing phages Gamma and Cherry, and *B. subtilis* phages SPO1 and CampHawk, respectively.

### Single gene product analysis mirrors whole genome/proteome analysis

In addition to using whole genome or proteome comparisons to determine phage cluster assignment we recently demonstrated the utility of single gene product analysis using the mycobacteriophage tape measure protein (TMP) and major capsid protein (MCP) gene products [23]*. W*e were unable to use either TMP or MCP for *Bacillus* phage single-gene comparison because podoviruses do not have a TMP and the MCP was not reported or identified by a TBLASTN search for several of the 83 *Bacillus* phages (including clusters C, D and H). Three genes are thought to be common to all tailed phages, the MCP (the major constituent of the icosahedral shell), portal protein (forms the pore into the capsid through which the DNA is packaged) and large terminase (the ATPase that packages the DNA into capsid) [24]. A putative large terminase gene product (TerL) was identified in 100% of the *Bacillus* phages and was, therefore, used for single-gene comparison (Figure 4). A dot plot alignment of the terminase gene products (TerL) confirmed our basic subcluster/cluster assignment with 100% of phages grouping by their pre-assigned subclusters and 90% by their clusters, while 12 of 15 singletons remaining singletons. Cluster B phage BceA1 was the only phage that appeared to have a terminase that was not homologous to the rest of its cluster. This overall percentage (95.2%) is comparable to the 98.8% reported for the mycobacteriophages using TMP [23]*.* The terminase dot plot analysis is supported by a neighbor-joining tree in which all of the proteins grouped by cluster/subcluster, with the exception of BceA1 and six of the 15 singletons which associated with another cluster (Figure 5). The few outliers are consistent with a recent analysis that suggested genes encoding TerL have undergone sufficient horizontal transfer between phage groups to disrupt some correlations between terminase sequence type and cluster relationship [25].

**Figure 4:**
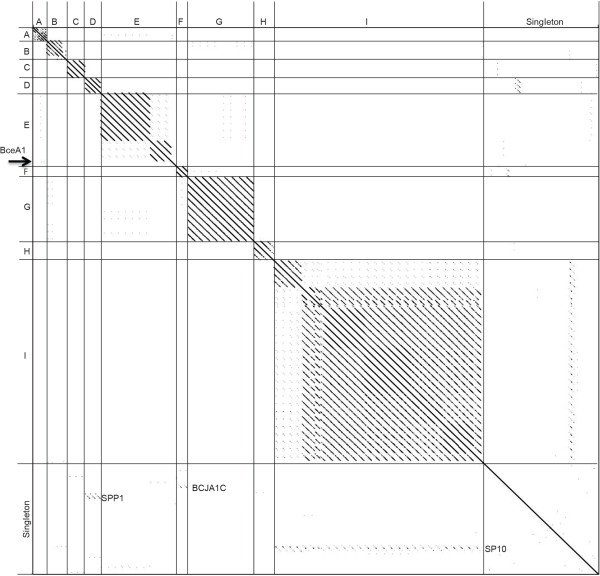
**Single gene amino acid dot plot analysis using the large terminase mirrors whole genome cluster assignment of** ***Bacillus*** **phages.** *Bacillus* phage clusters A-J are indicated on both the X-and Y-axis. Sequences for comparison were chosen by annotated large terminase gene products or a BlastP alignment to the closest relative when unannotated. Dot plots were produced using Gepard [57].

**Figure 5:**
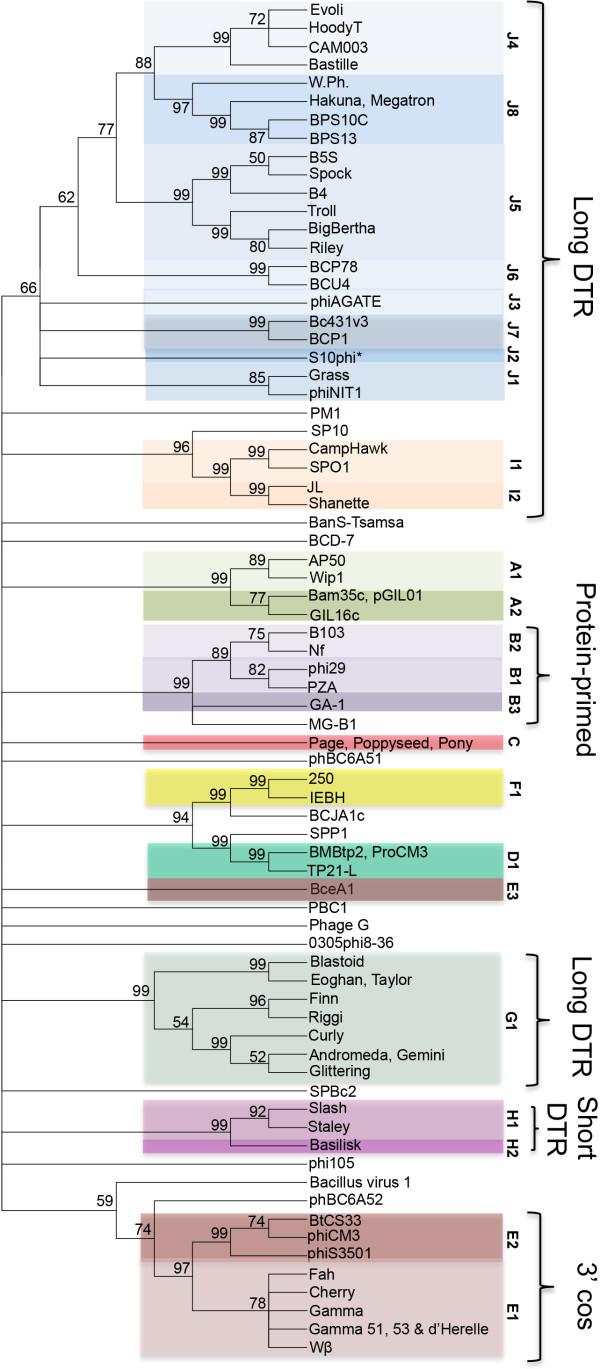
**A neighbor-joining tree analysis of the** ***Bacillus*** **terminase mirrors whole genome cluster assignments.** Phage names are colored by whole genome subcluster assignment, and this subcluster assignment is indicated on the right. Putative replication strategies for phages are also indicated when known. Abbreviations are direct, terminal repeats (DTR) and cohesive ends (cos). The phylogenetic tree was constructed using a MUSCLE [58] alignment and the neighbor-joining method in Mega5 [59]. Bootstrapping was set to 2000 and the unrooted tree was collapsed at a less than 50% bootstrap value.

From single-gene comparison, one of the subclusters appears to be unrelated to the rest of the cluster in which it belongs (subcluster B3 phage BceA1) while six singletons display similarity to other terminases. The SP10 terminase is similar to those in cluster I (SPO1-like), MG-B1 is similar to those in cluster B (Phi 29 -like), SPP1 and BCJA1c terminases are similar to those of clusters D (TP21L-lke) and F (IEBH-like), while Bacillus virus 1 and phBC6A52 display remarkable similarity to terminases of the E cluster (Gamma d’Herelle-like). These relationships could indicate more distant/ancient relationships over the entire chromosome or small regions of genetic exchange. The limited similarity of BceA1 TerL proteins to the rest of the B cluster is consistent with its distant whole genome/proteome relationships (faint diagonal lines on both the nucleotide and amino acid dot plots, see Figure 1). Phages SP10 and MG-B1 also show significant overall similarity to the I (SPO1-like) and B clusters (Phi 29-like), respectively (see supercluster discussion below for the SP10/cluster I relationship). Very weak similarity between B cluster phages and phage MG-B1 appears in dot plots and the similarity of MG-B1 to the phi 29 family was previously reported by Redondo et al. [26]. CoreGenes analysis and genome mapping indicates 11 MG-B1 gene products in common with the entire B cluster (29% of the proteome), and they are found in the same order (Figure 6), however, 7,475 bp larger than the rest of cluster B (30% larger), containing an extra 15–25 gene products by CoreGenes analysis. Further phage isolation will most likely deduce its precise relationship.

**Figure 6:**
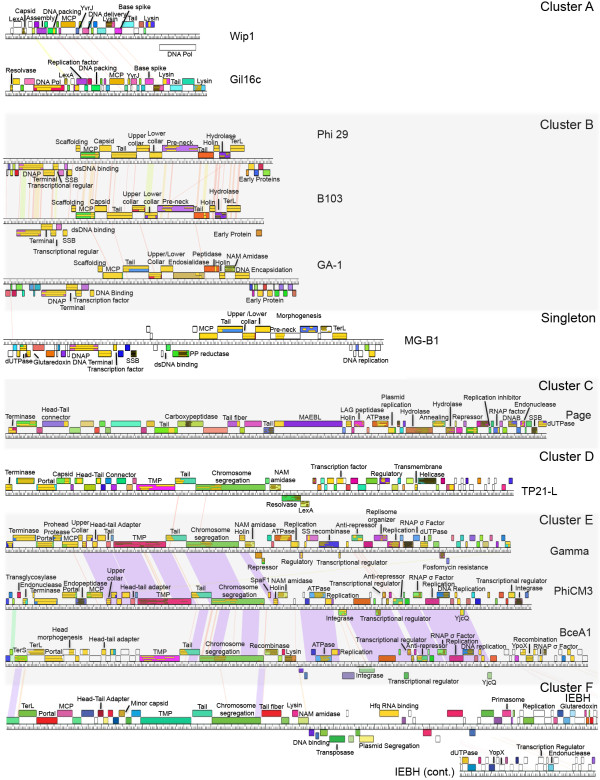
**A comparison of gene content and order within the** ***Bacillus*** **phage clusters reveals modularity and great diversity.** Genome maps for representative phages from the subclusters within *Bacillus* phage clusters A-F are provided along with singleton MG-B1. Phages were mapped using Phamerator [34], where purple lines between phages denote regions of high nucleotide similarity and the ruler corresponds to genome base pairs. Boxes for gene products are labeled with predicted function, occasionally numbered, and colored to indicate similarity between the phages (E-value <1e - 4). Abbreviations are adenosine triphosphatase (ATPase), DnaB helicase (DNAB), double-stranded DNA binding (dsDNA binding), 2’-deoxyuridine 5’-triphosphatase (dUTPase), major capsid protein (MCP), N-acetyl-muramyl-L-alanine amidase (NAM amidase), pyrophosphate reductase (PP reductase) RNA polymerase (RNAP), sigma factor (σ factor), large terminase (TerL), small terminase (TerS), tape measure protein (TMP), pilus specific protein, ancillary protein involved in adhesion (SpaF1), single-stranded binding protein (SSB), single-strand recombinase (SS recombinase).

Weaker relationships are displayed by BCJA1, Bacillus virus 1 and phBC6A52. Phage BCJA1c shares only 14-22% of its proteome with cluster D and F phages, while Bacillus virus 1 and phBC6A52 share only 10-22% of their proteome with phages in cluster E. In contrast, CoreGenes analysis suggests only small regions of genetic exchange for SSP1 in that it shares only ~5% of its proteome with the cluster D/F phages (including the terminase, tailspike, DnaB/DnaD replication protein, and the single stranded DNA binding and annealing proteins).

### Predicting phage replication strategies by terminase conservation

The identification and analysis of *Bacillus* phage terminase proteins presented in Figures 4 and 5 can also provide valuable insight into the replication strategy of these highly uncharacterized phages by comparing their terminases to those of well-characterized phages. Such comparisons have been used to determine the replication strategy of phages that infect diverse hosts such as *Enterobacteriaceae* and *Paenibacillus larvae*[14, 27]. In our analysis, several *Bacillus* phages contain terminases that were similar to the well-characterized SPO1 *Bacillus* phage, suggesting that they replicate and package their DNA by a similar concatemer strategy resulting in non-permuted DNA with long, direct terminal repeats [28, 29]. The cluster I phages had terminases of at least 87% similarity to SPO1 by BLASTP, while clusters G and J were weakly similar (~43% and ~56% similar, respectively) and singleton phage SP10 was 68% similar. Cluster E, phBC6A52 and Bacillus virus 1 terminases have weak homology to the HK97 terminase (42%- 45% similarity) which packages by 3’ cos ends, while phages of cluster H and singleton BanS-Tsamsa may have short DTRs due to weak homology to the *Clostridium* phage C terminase (~47% similarity) [30]. The B cluster phages and singleton MG-B1 have terminases that are homologues of the phi 29 terminase, suggesting they replicate DNA with a similar protein-primed replication strategy [31].

### Identification of two superclusters describing distantly related phages through proteome conservation analysis

In an effort to identify more distantly related phages belonging to “superclusters”, we carefully analyzed faint nucleotide and proteome dot plot lines, CoreGenes percentages, and whole genome maps for intercluster relationships. The genomic map of a representative phage from each subcluster is given in Figure 6 as an example, however the larger phages are excluded due to space constraints (clusters A through F are shown). Since short regions of similarity are common among phages, phages had to have similarity in genome content and order (synteny) to be termed a supercluster. Table 3 lists the two superclusters identified in this analysis.

**Table 3:** ***Bacillus*** **phage superclusters describe distantly related phages sharing significant proteome conservation**

Supercluster	Phages	% proteome conserved*
Gamma d’Herelle like	Clusters D, E and F, phage PBC1	~21% (D, E and F)
		~32% (F and PBC1)
SPO1-like	Clusters I, Cluster J	~27%

*Percent proteome conserved is the percentage conserved between two phages within different clusters as determined by CoreGenes.

Faint lines can be seen in both the nucleotide and proteome dot plots between clusters D, E and F as well as singleton PBC1. In addition, a similar genome content and order can be seen between these phages (for example phages TP21-L, Gamma and IEBH) where the first section of the chromosome contains phage structure and assembly genes and the last section harbors DNA metabolism genes (see Figure 6). These clusters also share an appreciable percentage of their proteome, with cluster D, E and F phages sharing ~21% of their proteome with at least two members of another cluster. This observation suggests an ancient relationship that has diverged. Singleton PBC1 also shares 32% of its proteome with the cluster F phages. These proteins include the portal protein, the MCP, three putative minor capsid proteins, a putative minor structural protein, the TMP, a holin, a glutaredoxin-like protein and nine hypothetical proteins. The environmental success of gamma-like phages is well documented (for a recent review see [32]). We have grouped the clusters D, E and F together with singleton PBC1 as the gamma d’Herelle-like supercluster, named after this well-characterized phage.

Clusters I, J and singleton SP10 have similar relationships, with I and J cluster phages sharing up to 27% of their proteome. Singleton SP10 shares ~29% of its proteome with cluster I phages and ~24% with cluster J phages, including several structural proteins (portal protein, MCP, minor structural protein, tail sheath, tail tube, tail assembly chaperone, tail lysin, tail fiber, tail baseplate and tail spike proteins), DNA replication proteins (DNA helicases, primase, endonuclease, exonuclease, and ribonuclotide reducatase), a peptidoglycan binding protein, a tRNA processing protein, several RNA polymerase sigma factors, and hypothetical proteins. Of interest, phage SP10 had previously been described as a SPO1-related phage by its discoverers [33]. This supercluster comprised of clusters I, J and singleton SP10 is termed the SPO1-like supercluster after these well characterized phages with family members that can infect many genera [29].

### DNA metabolism, cell lysis, structural, and host gene products are well conserved in Bacillus phages

Phamerator [34] was used to determine the most highly conserved gene products within the 83 fully sequenced *Bacillus* phages, and the extent of conservation among the phages. Phamerator identified a total of 4,442 phams, or groups of proteins with homology to one another. Of these, 894 (20%) had a predicted function and 3,548 (80%) were uncharacterized. In addition, 2,583 (58%) were orphams (phams containing a single member). This analysis confirms the highly diverse and uncharacterized nature of the *Bacillus* phages and underscores the immense biological reservoir that is present. Table 4 (phams with predicted function) and Table 5 (phams with uncharacterized proteins) contain the highly conserved phams that have twenty or more members. These phams are partitioned by their function as DNA replication/metabolism proteins, virion structure and assembly proteins, cell lysis proteins, or proteins involved in gene expression or host function. It is important to note that there may be other proteins with similar function not included in a pham due to lack of sufficient homology.

**Table 4:** **Common, conserved** ***Bacillus*** **phage proteins of predicted function with at least 20 homologues**

Pham #	Domain/Function	# Members	# Phages	Phages (cluster or phage name)
**DNA REPLICATION/METABOLISM**				
236	DNA Polymerase I	41	26	I, J1, J2, J3, J4, J5, J6, J8, SP10, Bc431v3
274	Chromosome Seg. ATPase	38	25	H2, J, BanS-Tsamsa
247	Class I RNR	36	33	H, I1, J, 0305φ836, BCD7, SP10, BanS-Tsamsa, SPB2
261	Helicase	28	28	I, J, SP10
256	SbcC-like Nuclease	28	28	I, J, SP10
257	Endonuclease (SbcD)	27	27	I, J1, J2, J3, J4, J5, J6, J7, BPS10C, SP10, W.Ph., Hakuna, Megatron
101	Dihydrofolate Reductase	27	27	H2, J, BCD7, BanS-Tsamsa, G
98	Thymidylate Synthase	24	24	J, BCD7
99	dNMP Kinase	23	23	J
195	Fumerate Reductase	22	23	J
222	Mre11 Nuclease	23	23	J
232	DNA Polymerase III	23	23	J
238	Histone	23	23	J
252	DUT	23	23	J
258	Replicative DNA Helicase	23	23	J
229	RecA-like	23	23	J
254	DNA primase	23	23	J
246	RNR beta subunit	22	22	H1, I1, J4, J5, J6, J8, 0305phi8-36
33	Chromosome Seg. Protein	22	22	J1, J2, J4, J5, J6, J7, J8
244	Glutaredoxin-like	20	19	J1, J2, J4, J5, J6, J8, SPBc2, BPS10c, BPS13, Megatron, W.Ph
**VIRION STRUCTURE AND ASSEMBLY**				
278	Tail Assembly Chaperone	46	23	J
89	Phage Terminase Large	34	28	I, J, SP10
264	Adsorption Tail	29	27	I, J1, J3, J4, J5, J6, J7, J8
291	MCP	29	28	I, J, SP10
276	Tail Lysin	29	28	I, J, SP10
295	Portal protein	28	28	I, J, SP10
266	Baseplate	28	28	I, J, SP10
283	Structural Protein	28	28	I, J, SP10
284	Tail Sheath	28	28	I, J, SP10
293	Prohead Protease	28	27	I, J2, J3, J4, J5, J6, J7, J8, phiNIT1, SP10
265	Baseplate J	27	23	J
273	Tail Lysin	27	24	J, BanS-Tsamsa
277	Major Capsid L1 Protein	24	24	J, SP10
88	Terminase	23	23	J
**CELL LYSIS PROTEINS**				
276	Cell Wall Hydrolase	28	28	I, J, SP10
282	Murein Transglycosylase	28	28	I1, J, 0305φ8-36, SP10, BanS-Tsamsa
226	Holin	23	23	J
198	Holin	23	23	J
267	Lysozyme-like	23	23	J
**GENE REGULATION/HOST FUNCTIONS**				
35	Bacterial SH3-like	25	25	C, E1, F, J5, J8, BanS-Tsamsa, PhiCM3
370	FtsK DNA Translocase	23	23	C, E1, G, BV1, PM1, phIS3501, phBC6A51
260	cAMP Regulatory Protein	23	23	J
269	MisL-like transporter	23	23	J
292	Membrane Protein	23	23	J
155	Metallo-beta-lactamase	21	20	J4, J5, J6, J7, J8, BanS-Tsamsa

Abbreviations include deoxynucleotide monophosphate kinase (dNMP kinase), chromosome segregation adenosine triphosphatase (chromosome seg ATPase), ribonucleotide diphosphate reductase (RNR), deoxyuridine nucleotidylhydrolase (DUT), and major capsid protein (MCP). Gene products are given and are organized by basic function (DNA Replication/Metabolism, Virion Structure and Assembly, Cell Lysis Proteins or Gene Regulation/Host Functions).

*Pham #’s are specific to this analysis due to assignment by Phamerator [34].

**Table 5:** **Common, conserved** ***Bacillus*** **phage proteins of uncharacterized function with over 20 homologues**

Pham #	# Members	# Phages	Phages (cluster or phage name)
			**Uncharacterized Proteins**
248	28	28	I, J, SP10
289	28	28	I, J, SP10
263	24	24	I1, J1, J3, J4, J5, J6, J7, J8
288	24	23	J
235	23	23	J
268	23	23	J
269	23	23	J
272	23	23	J1, J2, J4, J5, J6, J7, J8, BanS-Tsamsa
92	23	23	J
194	23	23	J
208	23	23	J
239	23	23	J
286	23	23	J
227	23	23	J
302	23	23	J
304	23	23	J
200	22	22	J1, J2, J3, J4, J5, J6, J7, Hakuna, Megatron, BPS10C, W. Ph.
225	22	22	J1, J2, J4, J5, J6, J7, J8
228	22	22	Grass, J2, J3, J4, J5, J6, J7, J8
255	22	22	J1, J2, J4, J5, J6, J7, J8
287	22	22	J1, J2, J3, J4, J5, J6, J7, Hakuna, Megatron, BPS10C, W. Ph.
290	22	22	J1, J2, J4, J5, J6, J7, J8
296	22	22	J1, J2, J3, J4, J5, J6, J7, Hakuna, Megatron, BPS10C, W. Ph.
301	22	22	Grass, J2, J3, J4, J5, J6, J7, J8
97	20	20	J3, J4, J5, J6, J7, J8
122	20	20	J3, J4, J5, J6, J7, J8

*Pham #’s are specific to this analysis due to assignment by Phamerator [34].

### DNA replication/metabolism

The most highly conserved *Bacillus* gene product is a class I ribonucleotide reductase (RNR, pham 247), with homologs found in 33 of the 83 phages and four phages have multiple homologs. RNR forms deoxyribonucleotides from ribonucleotides for DNA biosynthesis and is commonly found in lytic phages [35]. Other well-conserved proteins for nucleotide metabolism include a dihydrofolate reductase (conserved in 27 phages), thymidylate synthase (conserved in 24 phages), deoxynucleotide monophosphate kinase (conserved in 23 phages), fumerate reductase (conserved in 23 phages), deoxyuridine diphosphatase (DUT, conserved in 23 phages), RNR beta subunit (conserved in 22 phages) and a glutaredoxin-like protein (conserved in 22 phages). Many putative proteins involved in DNA replication and recombination were also identified including a DNA helicase (conserved in 28 phages), DNA exonuclease and endonuclease (conserved in 28 and 27 phages, respectively), DNA polymerase (conserved in 26 phages), two chromosome segregation proteins (conserved in 25 and 22 phages), and a Mre11-like nuclease, replicative helicase, DNA polymerase III, RecA homolog and DNA primase (each conserved in 23 phages). These results underscore the vital nature of efficient nucleotide metabolism in the propagation of lytic phages.

### Virion structure and assembly proteins

The structural and assembly proteins of the virion are also highly conserved gene products within the *Bacillus* phages, with phams consisting of a MCP, large terminase, portal protein, capsid structural protein, baseplate, tail sheath, and a tail lysin all having homologs in 28 of the 83 phages (34%). In addition, a procapsid protease, tail adsorption protein, tail lysin, virion structural protein, baseplate and another terminase have homologs in at least 23 of the 83 phages. These structural proteins are conserved among phages that are known myoviruses and siphoviruses, although the podoviruses and tectiviruses should also contain an MCP, portal protein and terminase. As discussed above, we were able to identify a large terminase for all of the *Bacillus* phages, meaning that these gene products had homologues that were somewhat characterized, but not homologous to the prevalent Pham. In contrast, we were unable to identify an MCP for many of the *Bacillus* phages, suggesting that homologs have not been described and emphasizing the need for further characterization of *Bacillus* phages. In support of this finding, recent studies have shown that MCP’s bearing no amino acid sequence similarity can harbor similar folds [36–40] hampering identification by sequence alone.

### Cell lysis

Cell lysis proteins are vital to the phage lifecycle, allowing them to exit the cell and infect other hosts. Five cell lysis proteins were well conserved including a cell wall hydrolase and murein-transglycosylase (each conserved in 28 phages), two holins (each conserved in 23 phages) and a lysozyme-like protein (conserved in 23 phages).

### Host functions/pathogenesis

Several gene products that are likely to regulate host functions were also highly conserved in *Bacillus* phages. A protein containing a bacterial SH3-like domain was identified in 25 of the 83 phages, including phages from cluster C, E, F, and J as well as the singletons phiCM3 and BanS-Tsamsa. The function of this protein is unknown but the SH3 domain is thought to mediate the assembly of large multiprotein complexes [41]. In addition, the cAMP regulatory protein (CRP) is found in 23 phages that may be used to control the expression of host carbon metabolism genes, which can contribute to bacterial virulence [42]. An FtsK/SpoIIIE-like cell division protein (gp22 in phage Cherry) was conserved in 23 of the phages (pham 370). This protein may control host transition into the sporulation state, contributing to the environmental fitness of *B. anthracis*[43]. As discussed above, pham 252 contains 23 DUT homologues, which are common in many bacteriophages and have been shown to function as G protein-like regulators required for the transfer of staphylococcal virulence factors [44, 45].

There are several other proteins that are less conserved that may contribute to host pathogenesis. Five *Bacillus* phages (SPO1, CampHawk, Pegasus, JL, and Shanette), encode a Pho-H like protein that aids in bacterial survival under phosphate starvation [46, 47]. Genes belonging to the phosphate regulon are reportedly very common in marine phages (40%) while they are less common in non-marine phages (4%) [48], in good agreement with our identification of PhoH in 5.4% of the *Bacillus* phages.

Subcluster E1 phages encode resistance to the soil antibiotic fosfomycin, which may account for the resistance reported for *B. anthracis* strains [43]. In addition, JL and Shanette both encode the tellurium resistance proteins TerE and TerC. Tellurium oxyanion (TeO_3_^2-^) has been used in the treatment of mycobacterial infections and resistance is a feature of many pathogenic bacteria. In fact, resistance is commonly used for the identification and isolation of Shiga toxin-producing *E. coli*[49].

### The comparison of subcluster and bacterial host reveals evolutionary boundaries

The *Bacillus* hosts in this study can be assembled into two separate groups by relatedness, and this evolutionary boundary may define phage boundaries and predict barriers for pathogenic gene transfer. *B. subtilis*, *B. megaterium* and *B. pumilus* are more closely related to each other than they are to the *Bacillus cereus* group of bacteria, comprised of *B. cereus*, *B. anthracis*, *B. thuringiensis*, *B. weihenstephanensis, B. mycoides* and *B. pseudomycoides*[50, 51]. To determine if there are such boundaries between phages and their hosts, the host from which each phage was isolated was compared within each cluster and subcluster.

The cluster to bacterial host relationship was somewhat ambiguous, with 70% of clusters populated by phages from only closely related *Bacillus* species (clusters A, B, C, D, E, F, and G) and others (clusters H, I and J) harboring phages from more distantly related *Bacillus* species (see Table 2). However, within these latter clusters there is a clear division at the subcluster level in that *B. subtilis*, *B. pumilus,* and *B. megaterium* phages *always* fall into a separate subcluster than phages that infect *B. cereus*, *B. thuringiensis*, *B. anthracis,* and *B. weihenstephanensis*. In fact, 22 of the 24 subclusters (92%) are divided by species, even when the cluster contains closely related species (the exceptions are subclusters J5 and J8, but these have closely related species). More phages are clearly needed to understand the host diversity within clusters, however, because only four clusters contain phages from diverse hosts (phages from both a *B. subtilis*, *B. pumilus*, or *B. megaterium* host and from a *Bacillus cereus* group host). In addition, this analysis was performed using only the host from which the phage was isolated since the host range of most of these phages is unknown. Host range studies will provide greater insight. For example, a recent finding that phage BPC78 inhibits both *B. cereus and B. subtilis* suggests that some phages are able to overcome this apparent host boundary [52].

The subcluster to host analysis also suggests a closer relationship between the *B. thuringiensis* and *B. cereus* species when compared to *B. anthracis*, since there is a subcluster division between *B. anthracis* phages and those that infect *B. thuringiensis* or *B. cereus* (see clusters A and E verses J). This apparent evolutionary separation is surprising given the recent report of five phages that infect *B. anthracis*, *B. thuringiensis* as well as the *B. cereus* host on which they were isolated (BanS-Tsamsa [53], Bc431v3 [54], and JL, Shanette, and Basilisk [40]).

## Conclusions

Phages are intimately linked to the ecology and evolution of their hosts, making characterization vital to understanding the diversity and evolution of the *Bacillus* genus. Herein we described the comparison of 83 fully sequenced *Bacillus* phages and their grouping into 10 clusters, 15 singletons and 24 subclusters (see Tables 1 and 2). In addition, two groups of more distantly-related phages were identified and termed “superclusters”, namely the SPO1-like and gamma d’Herelle-like. This analysis of *Bacillus* phages may aid in understanding newly isolated phages as well as the enormous complexity of tailed phages. It may also serve as a reference for comparisons to phages that infect other genera. Other such large-scale analyses are of 491 phages that infect *Mycobacterium* and of 337 phages that infect the *Enterobacteriaceae* family. Hatfull et al. grouped the Mycobacteriophages into ~17 “clusters” of related phages (A-Q) and 14 singleton clusters [13], while Grose and Casjens grouped the *Enterobacteriaceae* phages into 38 clusters of related phages and 18 singleton clusters [14]. In contrast to both of these phage groups, the *Bacillus* singletons outnumber the *Bacillus* clusters, presumably due to the decreased number of total phages isolated (83 phages as compared to 491 or 337). It should also be noted that additional *Bacillus* phage isolation will most likely require future revision of these cluster assignments as phages may be isolated that unite clusters.

Our analysis revealed several clusters of highly related phages (clusters C, D, F and G), and other clusters that contained very diverse phages (A, B, E, H, I, and J) (see Figures 2 and 3). Due to the low number of *Bacillus* phages isolated and the apparent expected diversity, it is currently unknown if these differences reflect differences in phage lifestyles, or if they occur due to sampling biases. Our analysis also revealed the need for using several analytical techniques to group phages, since one technique may suggest apparent relatedness that is weak by other techniques.

In addition to whole genome analysis, analysis of *Bacillus* phage gene products further underscores the enormity of *Bacillus* phage diversity, with 80% of protein phams (3,548) consisting of uncharacterized proteins. Because several phams of known function were identified that may contribute to host pathogenicity, understanding these uncharacterized phams is critical to understanding the evolution of pathogenic *Bacillus* strains.

The analysis of *Bacillus* phage evolutionary boundaries suggests that close phage relationships (defined by subclusters) are restricted by the relatedness of the host, with the phages that infect the *Bacillus cereus* group of phages more similar than those that infect *B. subtilis*, *B. megaterium* and *P. pumilus*. This analysis of host versus cluster is not only beneficial to understanding the evolution of *Bacillus* species but may indicate phage clusters more suitable for targeted phage therapy of pathogenic *B. cereus* and *B. anthracis* strains.

## Methods

### Computational analysis and genomic comparison

*Bacillus* phage sequences were retrieved from GenBank and the *Bacillus* Phage Database at PhagesDB.org as well as by contact with the authors of this website. To ensure retrieval of all *Bacillus* phages from GenBank, the major capsid protein (MCP) from at least one phage in each cluster was used to retrieve all phages with similar MCP sequence via TBLASTN [55]. Genomic maps of each phage were prepared using Phamerator [34], an open-source program designed to compare phage genomes. Phamerator was also used to calculate the percent G/C, number of ORFs and protein families or phams. The percentage of the proteome conserved was identified using the program CoreGenes 3.0 at the default BLASTP threshold of 75 [19, 20], while average nucleotide identity (ANI) was calculated by Kalign [56]. Dot plots were generated using Gepard [57]. For ease in dot plot analysis, long direct terminal repeats were removed from some phages, other phage genomes were reverse complemented, and new bp one calls were made to re-orient according to the majority of phages within a cluster. In addition, a portion of the PZA nucleotide sequence was reverse complemented to allow alignment with other phages of the cluster. Whole genome amino acid sequences were retrieved from Phamerator [34].

The terminase phylogenetic tree was constructed using a MUSCLE [58] alignment and the neighbor-joining method in Mega5 [59]. Bootstrapping was set to 2000 and the unrooted tree was collapsed at a less than 50% bootstrap value. Sequences for comparison were chosen by annotated large terminase gene products or a BlastP alignment to the closest relative when unannotated.

## Abbreviations

B: *Bacillus sp.*
T: *Tectiviridae*
P: *Podoviridae*
S: *Siphoviridae*
M: *Myoviridae*
MCP: Major capsid protein
TMP: Tape measure protein
ANI: Average nucleotide identity
bp: Base pair
kbp: Kilzobase pair
ORF: Open reading frame
pham: Phamerator
UK: Unknown.

## References

[1] Bergh O, Borsheim KY, Bratbak G, Heldal M (1989). High abundance of viruses found in aquatic environments. Nature.

[2] Brussow H, Hendrix RW (2002). Phage genomics: small is beautiful. Cell.

[3] Hambly E, Suttle CA (2005). The viriosphere, diversity, and genetic exchange within phage communities. Curr Opin Microbiol.

[4] Wilhelm SW, Jeffrey WH, Suttle CA, Mitchell DL (2002). Estimation of biologically damaging UV levels in marine surface waters with DNA and viral dosimeters. Photochem Photobiol.

[5] Wommack KE, Colwell RR (2000). Virioplankton: viruses in aquatic ecosystems. Microbiol Mol Biol Rev.

[6] Brovko LY, Anany H, Griffiths MW (2012). Bacteriophages for detection and control of bacterial pathogens in food and food-processing environment. Adv Food Nutr Res.

[7] Chan BK, Abedon ST, Loc-Carrillo C (2013). Phage cocktails and the future of phage therapy. Future Microbiol.

[8] Haque A, Tonks NK (2012). The use of phage display to generate conformation-sensor recombinant antibodies. Nat Protoc.

[9] Henry M, Debarbieux L (2012). Tools from viruses: bacteriophage successes and beyond. Virology.

[10] Murphy KC (2012). Phage recombinases and their applications. Adv Virus Res.

[11] Sharma M (2013). Lytic bacteriophages: Potential interventions against enteric bacterial pathogens on produce. Bacteriophage.

[12] Singh A, Poshtiban S, Evoy S (2013). Recent advances in bacteriophage based biosensors for food-borne pathogen detection. Sensors (Basel).

[13] Hatfull GF (2014). Mycobacteriophages: windows into tuberculosis. PLoS Pathog.

[14] Grose JH, Casjens SR (2014). Understanding the enormous diversity of bacteriophages: The tailed phages that infect the bacterial family Enterobacteriaceae. Virology.

[15] Hatfull GF, Jacobs-Sera D, Lawrence JG, Pope WH, Russell DA, Ko CC, Weber RJ, Patel MC, Germane KL, Edgar RH (2010). Comparative genomic analysis of 60 Mycobacteriophage genomes: genome clustering, gene acquisition, and gene size. J Mol Biol.

[16] Pope WH, Jacobs-Sera D, Russell DA, Peebles CL, Al-Atrache Z, Alcoser TA, Alexander LM, Alfano MB, Alford ST, Amy NE (2011). Expanding the diversity of mycobacteriophages: insights into genome architecture and evolution. PLoS One.

[17] Lee JH, Shin H, Ryu S (2014). Characterization and comparative genomic analysis of bacteriophages infecting members of the Bacillus cereus group. Arch Virol.

[18] Lorenz L, Lins B, Barrett J, Montgomery A, Trapani S, Schindler A, Christie GE, Cresawn SG, Temple L (2013). Genomic characterization of six novel Bacillus pumilus bacteriophages. Virology.

[19] Mahadevan P, King JF, Seto D (2009). Data mining pathogen genomes using GeneOrder and CoreGenes and CGUG: gene order, synteny and in silico proteomes. Int J Comput Biol Drug Des.

[20] Turner D, Reynolds D, Seto D, Mahadevan P (2013). CoreGenes3.5: a webserver for the determination of core genes from sets of viral and small bacterial genomes. BMC Res Notes.

[21] Pope WH, Anders KR, Baird M, Bowman CA, Boyle MM, Broussard GW, Chow T, Clase KL, Cooper S, Cornely KA (2014). Cluster M Mycobacteriophages Bongo, PegLeg, and Rey with Unusually Large Repertoires of tRNA Isotypes. J Virol.

[22] Pecenkova T, Paces V (1999). Molecular phylogeny of phi29-like phages and their evolutionary relatedness to other protein-primed replicating phages and other phages hosted by gram-positive bacteria. J Mol Evol.

[23] Smith KC, Castro-Nallar E, Fisher JN, Breakwell DP, Grose JH, Burnett SH (2013). Phage cluster relationships identified through single gene analysis. BMC Genomics.

[24] Casjens S, Hendrix R, Calendar R (1988). Control mechanisms in dsDNA bacteriophage assembly. The Bacteriophages.

[25] Casjens SR, Thuman-Commike PA (2011). Evolution of mosaically related tailed bacteriophage genomes seen through the lens of phage P22 virion assembly. Virology.

[26] Redondo RA, Kupczok A, Stift G, Bollback JP: **Complete Genome Sequence of the Novel Phage MG-B1 Infecting Bacillus weihenstephanensis.** *Genome Announcements* 2013.,**1**(3)**:** 10.1128/genomeA.00216-13 PMC3707571 23766400

[27] Casjens SR, Gilcrease EB (2009). Determining DNA packaging strategy by analysis of the termini of the chromosomes in tailed-bacteriophage virions. Methods Mol Biol.

[28] Klumpp J, Dorscht J, Lurz R, Bielmann R, Wieland M, Zimmer M, Calendar R, Loessner MJ (2008). The terminally redundant, nonpermuted genome of Listeria bacteriophage A511: a model for the SPO1-like myoviruses of gram-positive bacteria. J Bacteriol.

[29] Klumpp J, Lavigne R, Loessner MJ, Ackermann HW (2010). The SPO1-related bacteriophages. Arch Virol.

[30] Sakaguchi Y, Hayashi T, Kurokawa K, Nakayama K, Oshima K, Fujinaga Y, Ohnishi M, Ohtsubo E, Hattori M, Oguma K (2005). The genome sequence of Clostridium botulinum type C neurotoxin-converting phage and the molecular mechanisms of unstable lysogeny. Proc Natl Acad Sci U S A.

[31] Shih MF, Watabe K, Yoshikawa H, Ito J (1984). Antibodies specific for the phi 29 terminal protein inhibit the initiation of DNA replication in vitro. Virology.

[32] Gillis A, Mahillon J (2014). Phages preying on Bacillus anthracis, Bacillus cereus, and Bacillus thuringiensis: past, present and future. Viruses.

[33] Yee LM, Matsumoto T, Yano K, Matsuoka S, Sadaie Y, Yoshikawa H, Asai K (2011). The genome of Bacillus subtilis phage SP10: a comparative analysis with phage SPO1. Biosci Biotechnol Biochem.

[34] Cresawn SG, Bogel M, Day N, Jacobs-Sera D, Hendrix RW, Hatfull GF (2011). Phamerator: a bioinformatic tool for comparative bacteriophage genomics. BMC Bioinformatics.

[35] Dwivedi B, Xue B, Lundin D, Edwards RA, Breitbart M (2013). A bioinformatic analysis of ribonucleotide reductase genes in phage genomes and metagenomes. BMC Evol Biol.

[36] Parent KN, Gilcrease EB, Casjens SR, Baker TS (2012). Structural evolution of the P22-like phages: comparison of Sf6 and P22 procapsid and virion architectures. Virology.

[37] Rizzo AA, Suhanovsky MM, Baker ML, Fraser LC, Jones LM, Rempel DL, Gross ML, Chiu W, Alexandrescu AT, Teschke CM (2014). Multiple Functional Roles of the Accessory I-Domain of Bacteriophage P22 Coat Protein Revealed by NMR Structure and CryoEM Modeling. Structure.

[38] Shen PS, Domek MJ, Sanz-Garcia E, Makaju A, Taylor RM, Hoggan R, Culumber MD, Oberg CJ, Breakwell DP, Prince JT (2012). Sequence and structural characterization of great salt lake bacteriophage CW02, a member of the T7-like supergroup. J Virol.

[39] Zhang X, Guo H, Jin L, Czornyj E, Hodes A, Hui WH, Nieh AW, Miller JF, Zhou ZH (2013). A new topology of the HK97-like fold revealed in Bordetella bacteriophage by cryoEM at 3.5 A resolution. eLife.

[40] Grose JH, Belnap DM, Jensen JD, Mathis AD, Prince JT, Burnett SH, Breakwell DP (2014). The Genomes, Proteomes and Structure of Three Novel Phages that Infect the Bacillus cereus Group and Carry Putative Virulence Factors. J Virol.

[41] Kay BK (2012). SH3 domains come of age. FEBS Lett.

[42] Poncet S, Milohanic E, Maze A, Nait Abdallah J, Ake F, Larribe M, Deghmane AE, Taha MK, Dozot M, De Bolle X (2009). Correlations between carbon metabolism and virulence in bacteria. Contrib Microbiol.

[43] Schuch R, Fischetti VA (2006). Detailed genomic analysis of the Wbeta and gamma phages infecting Bacillus anthracis: implications for evolution of environmental fitness and antibiotic resistance. J Bacteriol.

[44] Tormo-Mas MA, Donderis J, Garcia-Caballer M, Alt A, Mir-Sanchis I, Marina A, Penades JR (2013). Phage dUTPases control transfer of virulence genes by a proto-oncogenic G protein-like mechanism. Mol Cell.

[45] Tormo-Mas MA, Mir I, Shrestha A, Tallent SM, Campoy S, Lasa I, Barbe J, Novick RP, Christie GE, Penades JR (2010). Moonlighting bacteriophage proteins derepress staphylococcal pathogenicity islands. Nature.

[46] Koonin EV, Rudd KE (1996). Two domains of superfamily I helicases may exist as separate proteins. Protein Sci.

[47] Kim SK, Makino K, Amemura M, Shinagawa H, Nakata A (1993). Molecular analysis of the phoH gene, belonging to the phosphate regulon in Escherichia coli. J Bacteriol.

[48] Goldsmith DB, Crosti G, Dwivedi B, McDaniel LD, Varsani A, Suttle CA, Weinbauer MG, Sandaa RA, Breitbart M (2011). Development of phoH as a novel signature gene for assessing marine phage diversity. Appl Environ Microbiol.

[49] Orth D, Grif K, Dierich MP, Wurzner R (2007). Variability in tellurite resistance and the ter gene cluster among Shiga toxin-producing Escherichia coli isolated from humans, animals and food. Res Microbiol.

[50] Maughan H, Van der Auwera G (2011). Bacillus taxonomy in the genomic era finds phenotypes to be essential though often misleading. Infect Genet Evol.

[51] Pilo P, Frey J (2011). Bacillus anthracis: molecular taxonomy, population genetics, phylogeny and patho-evolution. Infect Genet Evol.

[52] Lee JH, Shin H, Son B, Ryu S (2012). Complete genome sequence of Bacillus cereus bacteriophage BCP78. J Virol.

[53] Ganz HH, Law C, Schmuki M, Eichenseher F, Calendar R, Loessner MJ, Getz WM, Korlach J, Beyer W, Klumpp J (2014). Novel Giant Siphovirus from Bacillus anthracis Features Unusual Genome Characteristics. PLoS One.

[54] El-Arabi TF, Griffiths MW, She YM, Villegas A, Lingohr EJ, Kropinski AM (2013). Genome sequence and analysis of a broad-host range lytic bacteriophage that infects the Bacillus cereus group. Virol J.

[55] Altschul SF, Gish W, Miller W, Myers EW, Lipman DJ (1990). Basic local alignment search tool. J Mol Biol.

[56] Lassmann T, Sonnhammer EL (2005). Kalign–an accurate and fast multiple sequence alignment algorithm. BMC Bioinformatics.

[57] Krumsiek J, Arnold R, Rattei T (2007). Gepard: a rapid and sensitive tool for creating dotplots on genome scale. Bioinformatics.

[58] Edgar RC (2004). MUSCLE: multiple sequence alignment with high accuracy and high throughput. Nucleic Acids Res.

[59] Tamura K, Peterson D, Peterson N, Stecher G, Nei M, Kumar S (2011). MEGA5: molecular evolutionary genetics analysis using maximum likelihood, evolutionary distance, and maximum parsimony methods. Mol Biol Evol.

[60] Schuch R, Pelzek AJ, Kan S, Fischetti VA (2010). Prevalence of Bacillus anthracis-like organisms and bacteriophages in the intestinal tract of the earthworm Eisenia fetida. Appl Environ Microbiol.

[61] Sozhamannan S, McKinstry M, Lentz SM, Jalasvuori M, McAfee F, Smith A, Dabbs J, Ackermann HW, Bamford JK, Mateczun A (2008). Molecular characterization of a variant of Bacillus anthracis-specific phage AP50 with improved bacteriolytic activity. Appl Environ Microbiol.

[62] Verheust C, Fornelos N, Mahillon J (2005). GIL16, a new gram-positive tectiviral phage related to the Bacillus thuringiensis GIL01 and the Bacillus cereus pBClin15 elements. J Bacteriol.

[63] Stromsten NJ, Benson SD, Burnett RM, Bamford DH, Bamford JK (2003). The Bacillus thuringiensis linear double-stranded DNA phage Bam35, which is highly similar to the Bacillus cereus linear plasmid pBClin15, has a prophage state. J Bacteriol.

[64] Verheust C, Jensen G, Mahillon J (2003). pGIL01, a linear tectiviral plasmid prophage originating from Bacillus thuringiensis serovar israelensis. Microbiology.

[65] Paces V, Vlcek C, Urbanek P, Hostomsky Z (1986). Nucleotide sequence of the right early region of Bacillus subtilis phage PZA completes the 19366-bp sequence of PZA genome. Comparison with the homologous sequence of phage phi 29. Gene.

[66] Castilla-Llorente V, Salas M, Meijer WJ (2009). Different responses to Spo0A-mediated suppression of the related Bacillus subtilis phages Nf and phi29. Environ Microbiol.

[67] Gascon I, Lazaro JM, Salas M (2000). Differential functional behavior of viral phi29, Nf and GA-1 SSB proteins. Nucleic Acids Res.

[68] Khatemi BE, Chung On CC, Chamakura KR, Kuty Everett GF: **Complete Genome of Bacillus megaterium Podophage Pony.** *Genome Announcements* 2013.,**1**(6)**:** 10.1128/genomeA.00860-13 PMC3853050 24309727

[69] Lopez MS, Hodde MK, Chamakura KR, Kuty Everett GF: **Complete Genome of Bacillus megaterium Podophage Page.** *Genome Announcements* 2014.,**2**(2)**:** 10.1128/genomeA.00332-14 PMC3990757 24744341

[70] Klumpp J, Calendar R, Loessner MJ (2010). Complete Nucleotide Sequence and Molecular Characterization of Bacillus Phage TP21 and its Relatedness to Other Phages with the Same Name. Viruses.

[71] Dong Z, Peng D, Wang Y, Zhu L, Ruan L, Sun M: **Complete Genome Sequence of Bacillus thuringiensis Bacteriophage BMBtp2.** *Genome Announcements* 2013.,**1**(1)**:** 10.1128/genomeA.00011-12 PMC3569282 23405296

[72] Yuan Y, Gao M, Wu D, Liu P, Wu Y (2012). Genome characteristics of a novel phage from Bacillus thuringiensis showing high similarity with phage from Bacillus cereus. PLoS One.

[73] Fouts DE, Rasko DA, Cer RZ, Jiang L, Fedorova NB, Shvartsbeyn A, Vamathevan JJ, Tallon L, Althoff R, Arbogast TS (2006). Sequencing Bacillus anthracis typing phages gamma and cherry reveals a common ancestry. J Bacteriol.

[74] Minakhin L, Semenova E, Liu J, Vasilov A, Severinova E, Gabisonia T, Inman R, Mushegian A, Severinov K (2005). Genome sequence and gene expression of Bacillus anthracis bacteriophage Fah. J Mol Biol.

[75] Moumen B, Nguen-The C, Sorokin A (2012). Sequence Analysis of Inducible Prophage phIS3501 Integrated into the Haemolysin II Gene of Bacillus thuringiensis var israelensis ATCC35646. Genetics Res Int.

[76] Swanson MM, Reavy B, Makarova KS, Cock PJ, Hopkins DW, Torrance L, Koonin EV, Taliansky M (2012). Novel bacteriophages containing a genome of another bacteriophage within their genomes. PLoS One.

[77] Lee YD, Chang HI, Park JH (2011). Genomic sequence of temperate phage TEM126 isolated from wild type S. aureus. Arch Virol.

[78] Matthew SP, Decker SL, Chamakura KR, Kuty Everett GF: **Complete Genome of Bacillus pumilus Siphophage Glittering.** *Genome Announcements* 2013.,**1**(6)**:** 10.1128/genomeA.00856-13 PMC3853048 24309725

[79] Still EL, Riggi CF, Chamakura KR, Kuty Everett GF: **Complete Genome of Bacillus pumilus Siphophage Riggi.** *Genome Announcements* 2013.,**1**(6)**:** 10.1128/genomeA.00861-13 PMC3853051 24309728

[80] Mash SJ, Minahan NT, Chamakura KR, Kuty Everett GF: **Complete Genome of Bacillus pumilus Siphophage Blastoid.** *Genome Announcements* 2013.,**1**(6)**:** 10.1128/genomeA.00854-13 PMC3853047 24309724

[81] Hastings WJ, Ritter MA, Chamakura KR, Kuty Everett GF: **Complete Genome of Bacillus megaterium Siphophage Staley.** *Genome Announcements* 2013.,**1**(6)**:** 10.1128/genomeA.00864-13 PMC3853053 24309730

[82] Decrescenzo AJ, Ritter MA, Chamakura KR, Kuty Everett GF: **Complete Genome of Bacillus megaterium Siphophage Slash.** *Genome Announcements* 2013.,**1**(6)**:** 10.1128/genomeA.00862-13 PMC3861416 24336363

[83] Grose JH, Jensen JD, Merrill BD, Fisher JN, Burnett SH, Breakwell DP: **Genome Sequences of Three Novel Bacillus cereus Bacteriophages.** *Genome Announcements* 2014.,**2**(1)**:** 10.1128/genomeA.01118-13 PMC3900887 24459255

[84] Kropinski AM, Hayward M, Agnew MD, Jarrell KF (2005). The genome of BCJA1c: a bacteriophage active against the alkaliphilic bacterium, Bacillus clarkii. Extremophiles.

[85] Ritz MP, Perl AL, Colquhoun JM, Chamakura KR, Kuty Everett GF: **Complete Genome of Bacillus subtilis Myophage CampHawk.** *Genome Announcements* 2013.,**1**(6)**:** 10.1128/genomeA.00984-13 PMC3868849 24356825

[86] Kimura K, Itoh Y (2003). Characterization of poly-gamma-glutamate hydrolase encoded by a bacteriophage genome: possible role in phage infection of Bacillus subtilis encapsulated with poly-gamma-glutamate. Appl Environ Microbiol.

[87] Miller SY, Colquhoun JM, Perl AL, Chamakura KR, Kuty Everett GF: **Complete Genome of Bacillus subtilis Myophage Grass.** *Genome Announcements* 2013.,**1**(6)**:** 10.1128/genomeA.00857-13 PMC3853049 24309726

[88] Barylski J, Nowicki G, Gozdzicka-Jozefiak A (2014). The Discovery of phiAGATE, A Novel Phage Infecting Bacillus pumilus, Leads to New Insights into the Phylogeny of the Subfamily Spounavirinae. PLoS One.

[89] Lee JH, Shin H, Son B, Heu S, Ryu S (2013). Characterization and complete genome sequence of a virulent bacteriophage B4 infecting food-borne pathogenic Bacillus cereus. Arch Virol.

[90] Ting JH, Smyth TB, Chamakura KR, Kuty Everett GF: **Complete Genome of Bacillus thuringiensis Myophage BigBertha.** *Genome Announcements* 2013.,**1**(6)**:** 10.1128/genomeA.00853-13 PMC3853046 24309723

[91] Shin H, Lee JH, Park J, Heu S, Ryu S (2014). Characterization and genome analysis of the Bacillus cereus-infecting bacteriophages BPS10C and BPS13. Arch Virol.

[92] Boice LB (1969). Evidence that Bacillus subtilis bacteriophage SP02 is temperate and heteroimmune to bacteriophage phi-105. J Virol.

[93] Kong M, Kim M, Ryu S (2012). Complete genome sequence of Bacillus cereus bacteriophage PBC1. J Virol.

[94] Alonso JC, Luder G, Stiege AC, Chai S, Weise F, Trautner TA (1997). The complete nucleotide sequence and functional organization of Bacillus subtilis bacteriophage SPP1. Gene.

[95] Umene K, Shiraishi A (2013). Complete nucleotide sequence of Bacillus subtilis (natto) bacteriophage PM1, a phage associated with disruption of food production. Virus Genes.

[96] Lazarevic V, Soldo B, Dusterhoft A, Hilbert H, Mauel C, Karamata D (1998). Introns and intein coding sequence in the ribonucleotide reductase genes of Bacillus subtilis temperate bacteriophage SPbeta. Proc Natl Acad Sci U S A.

[97] Thomas JA, Hardies SC, Rolando M, Hayes SJ, Lieman K, Carroll CA, Weintraub ST, Serwer P (2007). Complete genomic sequence and mass spectrometric analysis of highly diverse, atypical Bacillus thuringiensis phage 0305phi8-36. Virology.

